# Chromosome-level reference genome of the Siamese fighting fish *Betta splendens*, a model species for the study of aggression

**DOI:** 10.1093/gigascience/giy087

**Published:** 2018-07-11

**Authors:** Guangyi Fan, Judy Chan, Kailong Ma, Binrui Yang, He Zhang, Xianwei Yang, Chengcheng Shi, Henry Chun-Hin Law, Zhitao Ren, Qiwu Xu, Qun Liu, Jiahao Wang, Wenbin Chen, Libin Shao, David Gonçalves, Andreia Ramos, Sara D Cardoso, Min Guo, Jing Cai, Xun Xu, Jian Wang, Huanming Yang, Xin Liu, Yitao Wang

**Affiliations:** 1 State Key Laboratory of Quality Research of Chinese Medicine, Institute of Chinese Medical Sciences, University of Macau, Avenida da Universidade, Taipa, Macau, China; 2 BGI-Qingdao, BGI-Shenzhen, Sino-German Ecopark, No.2877, Tuanjie Road, Huangdao District, Qingdao, Shandong Province, 266555, China; 3 BGI-Shenzhen, Building 11, Beishan Industrial Zone, Yantian District, Shenzhen 518083, China; 4 China National GeneBank, BGI-Shenzhen, Jinsha Road, Dapeng New District, Shenzhen 518120, China; 5 James D. Watson Institute of Genome Sciences, Hangzhou 310058, China; 6 Institute of Science and Environment, University of Saint Joseph, Rua de Londres 16, Macao SAR, China; 7 Instituto Gulbenkian de Ciência, Rua da Quinta Grande 6, 2780-156 Oeiras, Portugal

**Keywords:** *Betta splendens*, fish genome, aggression, Hi-C, chromosomal genome assembly, resequencing

## Abstract

**Background:**

Siamese fighting fish *Betta splendens* are notorious for their aggressiveness and accordingly have been widely used to study aggression. However, the lack of a reference genome has, to date, limited the understanding of the genetic basis of aggression in this species. Here, we present the first reference genome assembly of the Siamese fighting fish.

**Findings:**

Frist, we sequenced and *de novo* assembled a 465.24-Mb genome for the *B. splendens* variety Giant, with a weighted average (N50) scaffold size of 949.03 Kb and an N50 contig size of 19.01 Kb, covering 99.93% of the estimated genome size. To obtain a chromosome-level genome assembly, we constructed one Hi-C library and sequenced 75.24 Gb reads using the BGISEQ-500 platform. We anchored approximately 93% of the scaffold sequences into 21 chromosomes and evaluated the quality of our assembly using the high-contact frequency heat map and Benchmarking Universal Single-Copy Orthologs. We also performed comparative chromosome analyses between *Oryzias latipes* and *B. splendens*, revealing a chromosome conservation evolution in *B. splendens*. We predicted  23,981 genes assisted by RNA-sequencing data generated from brain, liver, muscle, and heart tissues of Giant and annotated 15% repetitive sequences in the genome. Additionally, we resequenced five other *B. splendens* varieties and detected ∼3.4 M single-nucleotide variations and  27,305 insertions and deletions.

**Conclusions:**

We provide the first chromosome-level genome for the Siamese fighting fish. The genome will lay a valuable foundation for future research on aggression in *B. splendens*.

## Data Description

Males of the Siamese fighting fish *Betta splendens* (NCBI:txid158456) are notorious for their aggressiveness. In nature, males establish and vigorously defend territories where they construct a bubble nest to hold fertilized eggs. In laboratory settings, males will readily attack an opponent, their mirror image, physical models of conspecifics, and video images of other males. Accordingly, the species has been widely used to study the neurobiological mechanisms of aggression. However, to date, the lack of a reference genome has limited studies on the genetic basis of aggression in *B. splendens*. The species is also one of the most relevant for the ornamental fish trade as it is easy to keep and reproduce in captivity. In addition, throughout its long domestication period, many varieties have been selected for their exuberant fins and colors, size, or aggressive behavior. Here, we sequenced the genome of *B. splendens* to provide the genomic foundation for future research on aggression and development of genomic tools.

### Sampling and sequencing

We purchased five varieties of adult male Siamese fighting fish, including Giant, Half-moon, Half-moon plakat, Fighter, and Elephant Ear from Hong Kong supplier TC Northern Betta for DNA and RNA extraction [1, 2] (Supplementary Fig. S1). We constructed and sequenced six DNA libraries for the *B. splendens* variety Giant, including three short insert size libraries and three mate-pair libraries (Supplementary Table S1), and five RNA-sequencing (RNA-seq) libraries (Supplementary Table S2) using the HiSeq 2000 sequencing platform. One Hi-C library for Giant was also constructed and sequenced using the BGISEQ-500 sequencing platform, yielding 75.24 Gb of reads. Additionally, we sequenced four short insert size DNA libraries for the other four *B. splendens* varieties.

### Genome assembly

**Figure 1:**
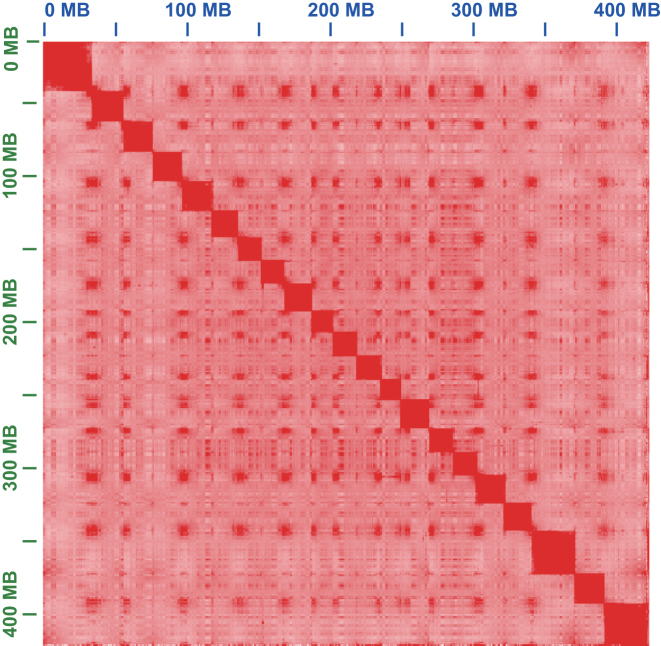
Hi-C interaction heat map for *B. Splendens* reference genome showing interactions between the 21 chromosomes.

We obtained 52.34 Gb of clean reads using SOAPnuke, version 1.5.3 (SOAPnuke, RRID:SCR_015025) [3], with strict parameters, including removal of low-quality reads, adapter contamination, and Polymerase chain reaction (PCR) duplicates. Then, we performed the *de novo* assembly of the Giant reads using SOAPdenovo2, version 2.04 (SOAPdenovo2, RRID:SCR_014986) [4], assembler. For the genome assembly, the short insert size libraries were used to construct the contig sequences and the mate-paired libraries were used to link the scaffolds. We filled the gaps within the scaffolds using GapCloser, version 1.12 (GapCloser, RRID:SCR_015026). We obtained a genome assembly with a size of 465.24 Mb, with an N50 scaffold size of 949.03 Kb and an N50 contig size of 19.01 Kb (Table 1), covering 99.93% of the estimated genome size of 465.55 Mb using kmer, version 1.0, analysis (Supplementary Table S3 and Fig. S2). To construct the reference genome at the chromosome level, we used a MBOI endonuclease to cut the DNA and constructed a Hi-C library based on a previous protocol [5]. We sequenced 75.24 Gb of data using the BGISEQ-500 sequencing platform and obtained 34.5 Gb valid reads (∼45.8%) that could be used to anchor the scaffolds into chromosomes after quality control using the HiC-Pro, version 2.8.0, pipeline [6, 7] (Supplementary Figs. S3–S7). Last, we constructed 21 chromosomes that occupied 95.3% of the genome (Fig. 1, Table 1, Supplementary Table S4) using Juicer [8], version 1.5, and 3D-dna, version 170123, pipeline [9] based on the draft genome assembly. To evaluate the quality of the assembly, we found 95.4% of Benchmarking Universal Single-Copy Orthologs (BUSCO), version 3.0.1 (BUSCO, RRID:SCR_015008), genes that could be completely covered by our genome (Table 2) and approximately 98% of the transcripts assembled from RNA-seq data that could be aligned against the genome with more than 90% coverage (Supplementary Table S5).

**Table 1:** Statistics of the assembly using SOAPdenovo and Hi-C data

Type	Scaffold original	Contig original	Scaffold (Hi-C)	Contig (Hi-C)
Total number	92,886	138,929	91,819	139,323
Total length (bp)	465,240,853	421,527,246	465,132,837	421,527,246
Average length (bp)	5,008.73	3,034.12	5,066	3,026
N50 (bp)	949,032	19,014	19,754,490	18,890
N90 (bp)	59,769	3,504	13,781,534	3,470

**Table 2:** Evaluation results of the genome and gene set using BUSCO

	Genome		Genes	
	Number	Percentage (%)	Number	Percentage (%)
Complete	4,375	95.4	4,128	90.1
Single-copy complete	4,232	92.3	3,937	85.9
Duplicated complete	142	3.1	191	4.2
Fragmented	128	2.8	338	7.4
Missing	82	1.8	118	2.5
Total	4,584	-	4,584	-

### Genome annotation

We annotated the repetitive sequences by combining *de novo* and homolog-based approaches [10]. First, we used LTR-FINDER, version 1.06 (LTR_Finder, RRID:SCR_015247) [11], and RepeatModeler, version 1.0.8 (RepeatModeler, RRID:SCR_015027), to construct a repetitive sequence library. Then, we used RepeatMasker, version 3.3.0 (RepeatMasker, RRID:SCR_012954) [12], to classify these repeat sequences. We also detected repetitive sequences using RepeatMasker and ProteinMasker, version 3.3.0, based on the Repbase library [13]. We identified 15.12% transposable elements in the genome (Supplementary Table S6).

For the protein-coding prediction, we combined the following approaches: gene model prediction using AUGUSTUS, version 3.0.3 (Augustus, RRID:SCR_008417) [14], and GENSCAN, version 1.0 (GENSCAN, RRID:SCR_012902) [15]; gene prediction using GeneWise, version 2.2.0 (GeneWise, RRID:SCR_015054) [16], based on the alignment results of protein sequences of other published species against our assembly; and five RNA-seq libraries were used to assist in predicting the gene structure with Cufflinks, version 2.2.1 (Cufflinks, RRID:SCR_014597) [17]. Last, we integrated all of this evidence into a nonredundancy gene set using GLEAN [18], version 1.0. The final gene set contained  23,981 genes (Supplementary Table S7), which is close to the number for *Oryzias latipes* [19] ( 24,674) and slightly less than that for *Danio rerio* [20] ( 26,046). We identified 90% of the 4,128 BUSCO gene models to be complete in the actinopterygii gene set (Table 2).

### Comparative genomic analysis

**Figure 2:**
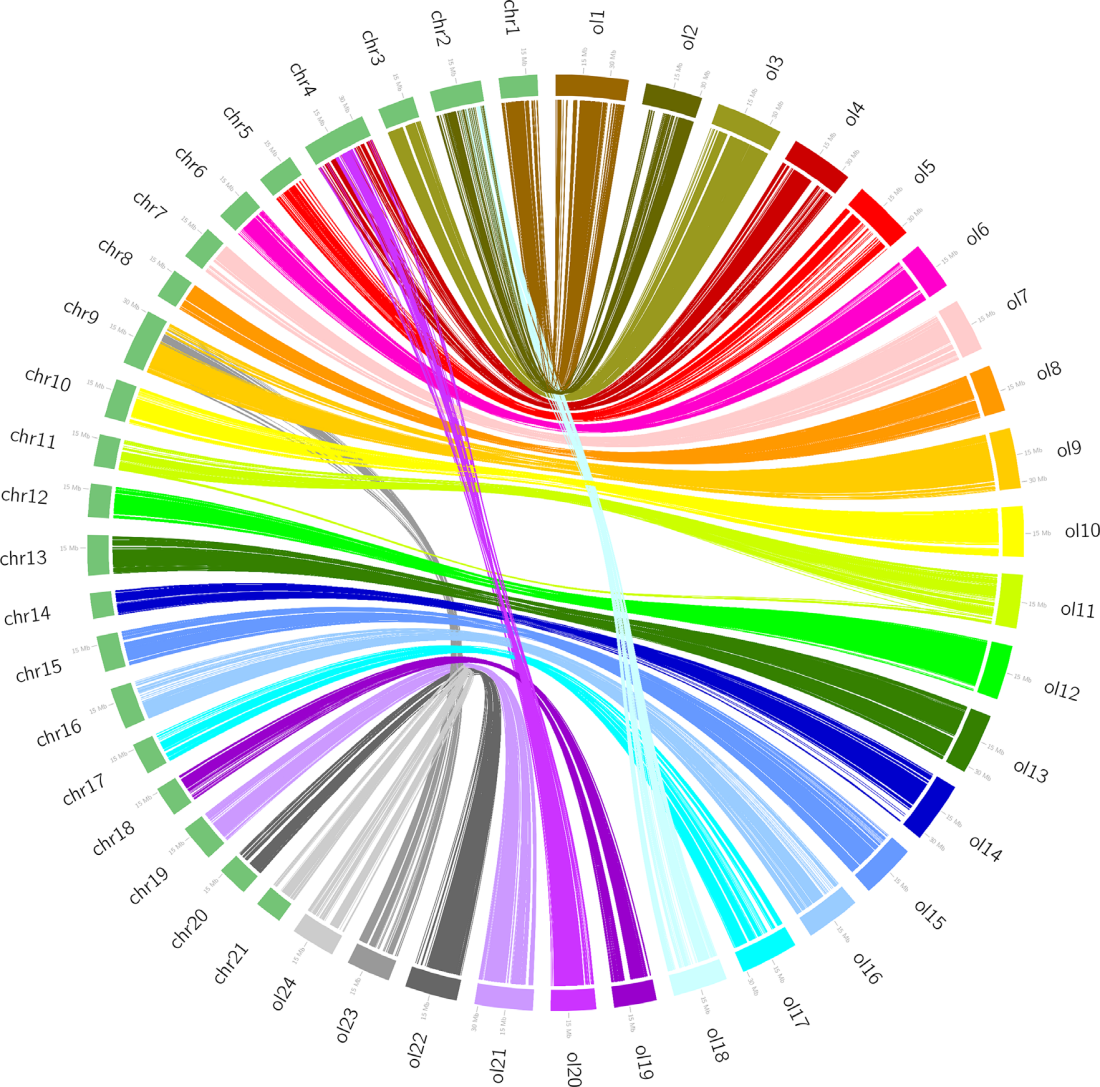
Collinear relationship between *B. splendens* and *O. latipes*. Green represents the chromosomes of *B. splendens* and the other multicolor represent the chromosomes of *O. latipes*.

**Figure 3:**
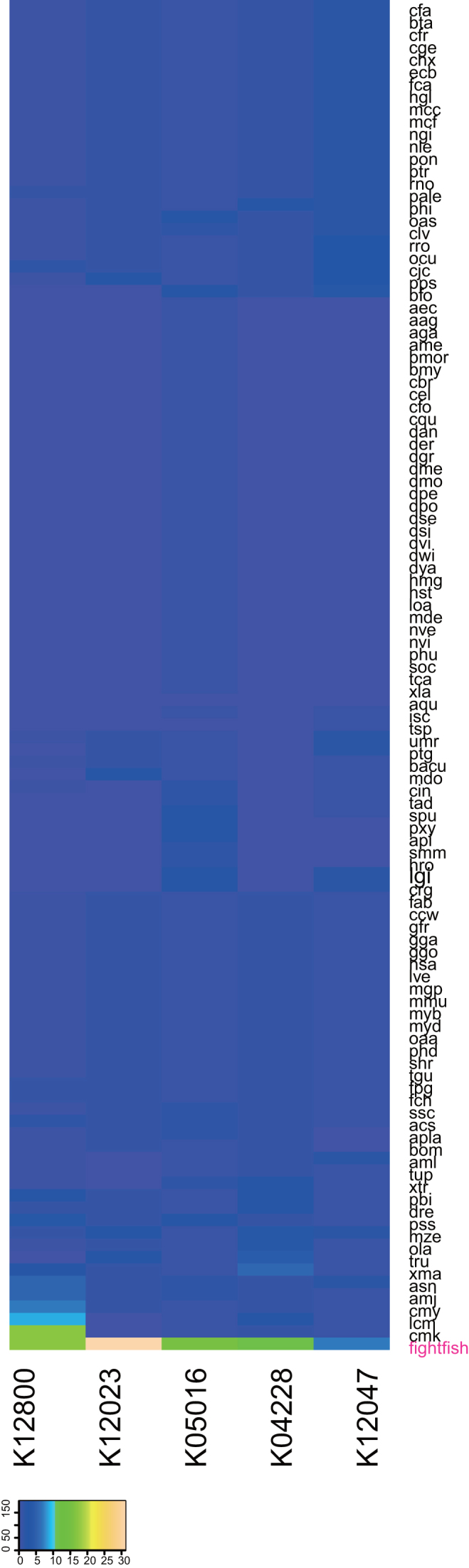
Five gene families with prominent expansion in *B. splendens* when compared with 109 other species including *NLRP3* (K12800), *TRIM47* (K12023), *CLCN7* (K05016), *AVPR2* (K04228), and *MGAM* (K12047) using the KEGG database.

**Figure 4:**
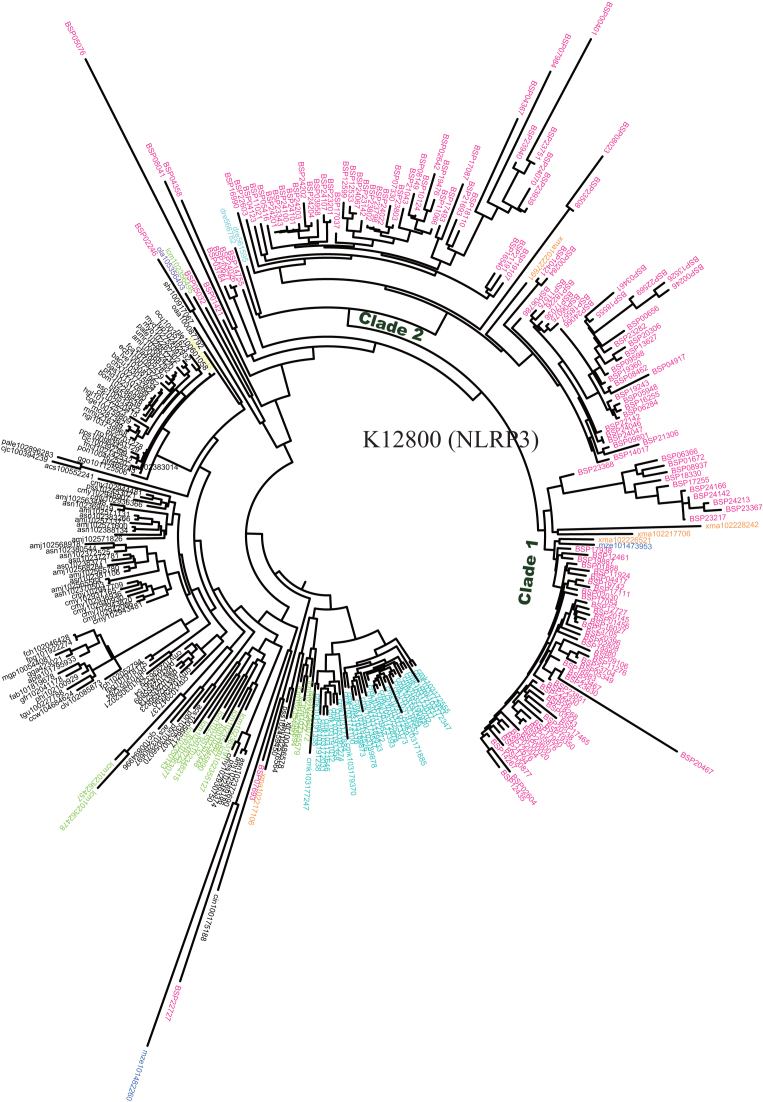
The gene phylogenetic tree of NLRP3 gene family (KO: K12800) using the genes of *B. splendens* and other species. Clade 1 and clade 2 show two prominent expansion subfamilies of *B. splendens*.

We compared the fighting fish genome with other species using Lastz, version 1.02.00, both at the whole-genome and gene levels. All of the 21 chromosomes assembled for the fighting fish could be matched to chromosomes of *O. latipes* with a mean coverage ratio of 75.3%. From these, 18 chromosomes had a single hit to one chromosome of *O. latipes*, and 3 chromosomes (1, 19, and 21) had a hit to two chromosomes of *O. latipes* (Fig. 2, Supplementary Table S8), indicating conservative evolution for most of chromosomes, as well as several chromosome reshuffling events between these two species. Furthermore, from the gene set level, KO (Kyoto Encyclopedia of Genes and Genomes [KEGG] Orthology) terms of animals from 109 species were counted and compared with the fighting fish gene set using the KEGG database [21], version 79. There were five KO terms notably expanded in fighting fish compared with all other animals, including 147 NACHT, LRR, and PYD domains-containing protein 3 (*NLRP3*, K12800), 86 tripartite motif-containing protein 47 (*TRIM47*, K12023), 43 chloride channel 7 (*CLCN7*, K05016), 29 arginine vasopressin receptor 2 (*AVPR2*, K04228), and 17 maltase-glucoamylase (*MGAM*, K12047) (Fig. 3). *NLRP3* has two prominent expansions, one corresponding to clade 1, containing 56 genes, and one corresponding to clade 2, containing 79 genes, whereas other fish species in these two clades have less than three gene copies (Fig. 4). *NLRP3* encodes a pyrin-like protein containing a pyrin domain, a nucleotide-binding site domain, and a leucine-rich repeat motif, and plays a role in the regulation of inflammation, the immune response, and apoptosis [22].

### Resequencing

Through mirror-image stimulation testing, we found that the different varieties of Siamese fighting fish have varying levels of aggressiveness. Male *B. splendens* were tested under a standardized mirror-elicited aggression paradigm as this elicits aggression levels similar to those of a real conspecific. One fighting fish was placed into the testing tank (30 × 19 × 23 cm) and left undisturbed for 30 minutes for acclimation. Then, the swimming behavior was recorded by taking a 5-minute video with a side digital camera; the swimming track was recorded using the Viewpoint ZebraLab Tracking System for 5 minutes. This represented the control state. After that, a mirror of similar size with a side wall was placed into the tank to induce aggression of the fish by its own mirror image. Aggression was observed through the following behaviors: opecular flare, fin spreading, 90° turn, and mirror hit. As expected, the mirror image elicited a high frequency of aggressive displays. Fish spent most of the time close to the mirror side and increased overall swimming distance compared to controls. Among all tested varieties, Giant had the highest frequency of aggressive displays and Half-moon had the lowest (Supplementary Fig. S8).

To evaluate the genetic diversity among the four varieties of *B. splendens*, we called the single-nucleotide variations (SNVs) and insertions and deletions (indels) based on the read alignment result using the Giant assembly as a reference. We obtained 70.25 Gb of clean reads filtered from 79.18 Gb of raw reads (Supplementary Table S9). We used BWA, version0.6.2 (BWA, RRID:SCR_010910) [23], to align all the resequencing data to the reference genome and the UnifiedGenotyper in Genome Analysis Toolkit, version2.8.1 (GATK, RRID:SCR_001876) [24], to call variations. In total, we detected approximately 3.4 M SNVs and 27,305 indels, which will provide a rich source of genomic data for use in future research and applications.

## Availability of supporting data

Data is deposited in NCBI under the BioProject accession number PRJNA416843. Supporting data are also available via the *GigaScience* database GigaDB [25].

## Additional files

**Supplementary Figure S1:** Five different varieties of adult male Siamese fighting fish.

**Supplementary Figure S2:** Distribution of the 17-mer analysis for the five Siamese fighting fish varieties.

**Supplementary Figure S3:** Quality control of Hi-C read alignment against genome sequence. Statistics for the type of separated pair-end read alignment. The aligned read ratio shown in the left bar including full read and trimmed read mapping.

**Supplementary Figure S4:** Quality control of read pairing. Considering the alignment type and read pairing, all paired reads include uniquely aligned pairs (Reported pairs), unmapped pairs (Unmapped pairs) and others (Not Reported pairs). The right bar shows the “Not Reported pairs”, which including low quality alignment, singleton and multiple hits.

**Supplementary Figure S5:** Statistic of read pair filtering. When assigned to restriction fragments, the aligned pairing reads can be divided to valid and invalid pairs. A valid paired-read involves two different restriction fragments and can be divided into four types according the direction of reads (the middle bar). F means Forward and R means Reverse. Invalid pairs content is shown in the right bar.

**Supplementary Figure S6:** Fraction of duplicated reads. The left bar shows the ratio of duplication for the valid read pairs. For all the non-duplicated reads, the percentage of cis and trans contacts are shown (right bar).

**Supplementary Figure S7:** Distribution of the fragment size. According to the distance between alignment site and the end of restriction fragment, a fragment size can be calculated.

**Supplementary Figure S8:** Evaluation of aggressive behavior in different varieties of *Betta splendens*. Number of opecular flare, mirror hit, 90° turning and fin spreading were counted manually from the 5-min video recording the swimming of normal and activated fish of different varieties. Data was expressed as mean ± S.D. of 6 replicates (n = 6).

**Supplementary Table S1:** Summary of sequencing data generated in this study.

**Supplementary Table S2:** Summary of the RNA-Seq data generated using the HiSeq 2000 sequencing platform.

**Supplementary Table S3:** 17-mer analysis information for the five Siamese fighting fish varieties.

**Supplementary Table S4:** Length of the 21 chromosomes constructed for the Siamese fighting fish Giant variety, given in descending order.

**Supplementary Table S5:** Assessment of the gene region coverage of assembly using RNA-seq data.

**Supplementary Table S6:** Statistics for the transposable element (TE) sequences present in the Siamese fighting fish genome.

**Supplementary Table S7:** Statistics of predicted gene models.

**Supplementary Table S8:** Summary of the alignment between *Betta splendens* and *Oryzias latipes*.

**Supplementary Table S9:** Summary of the resequencing data generated in this study.

## Abbreviations

indel: insertions and deletions; KO: Kyoto Encyclopedia of Genes and Genomes Orthology; RNA-seq: RNA sequencing; SNV: single-nucleotide variation.

## Author contributions

G.F., S.L., and J.C. conceived the project. G.F., X.L., and J.C. supervised the research. H.Z., C.S., and X.Y. conceived and designed the experiments. K.M., X.L., and B.Y. performed genome assembly and gene annotation. H.L., Z.R., Q.L., and Q.X. prepared the fighting fish sample. Jiahao. W., W.C., X.X., and L.S. performed sequencing. A.R., M.G., Jing. C., H.Y., and J.W. performed comparative genomic analysis. G.F., S.C., Y.W., and D.G. revised the paper. K.M. and X.Y. performed data accession.

## Supplementary Material

GIGA-D-18-00046_Original_Submission.pdf

GIGA-D-18-00046_Revision_1.pdf

GIGA-D-18-00046_Revision_2.pdf

Response_to_Reviewer_Comments_Original_Submission.pdf

Response_to_Reviewer_Comments_Revision_1.pdf

Reviewer_1_Report_(Original_Submission) -- Ole K Tørresen 3/13/2018 Reviewed

Reviewer_1_Report_Revision_1 -- Ole K Tørresen 6/14/2018 Reviewed

Reviewer_2_Original_Submission_(attachment).pdf

Reviewer_2_Report_(Original_Submission) -- Andrew Thompson 3/16/2018 Reviewed

Supplemental Tables and Figures
