# Supplementary material for: Chromosome-level reference genome of the Siamese fighting fish Betta splendens, a model species for the study of aggression
Source: Gigascience. 2018 Jul 11;7(11):giy087. doi: 10.1093/gigascience/giy087 (PMC6251983; doi:10.1093/gigascience/giy087)
Supplement: GIGA-D-18-00046_Original_Submission.pdf [file giy087_giga-d-18-00046_original_submission.pdf]

GigaScience

High-quality reference genome of the Siamese fighting fish Betta splendens, a model species for the study of aggression

--Manuscript Draft--

**Manuscript Number:**	GIGA-D-18-00046
**Full Title:**	High-quality reference genome of the Siamese fighting fish Betta splendens, a model species for the study of aggression
**Article Type:**	Data Note
**Funding Information:**	Macau Science and Technology Development Fund (011/2014/A1) — dr. Simon Ming-Yuen Lee<br>Shenzhen Municipal Government of China (JCYJ20151015162041454) — dr. Wenbin Chen<br>Shenzhen Municipal Government of China (JCYJ20150529150505656) — Dr. Xin Liu
**Abstract:**	Background: Siamese fighting fish Betta splendens are notorious for their aggressiveness and accordingly have been widely used to study aggression. However, the lack of a reference genome has so far limited the understanding of the genetic basis of aggression in this species. Here we present the first reference genome assembly of the Siamese fighting fish.<br>Findings: We first sequenced and de novo assembled a 465.24 Mb genome for the B. splendens variety Giant, with a weighted average (N50) scaffold size of 949.03 Kb and an N50 contig size of 19.01 Kb, covering 99.93% of the estimated genome size. To obtain a chromosome-level genome assembly, we constructed one Hi-C library and sequenced 69.7 Gb reads using the BGISEQ-500 platform. We anchored approximately 93% of the scaffold sequences into 21 chromosomes and evaluated the quality of our assembly using the high contact frequency heatmap and BUSCO. We also performed comparative chromosome analyses between Oryzias latipes and B. splendens, revealing a chromosome conservation evolution in B. splendens. We predicted a total of 23,202 genes assisted by RNA-seq data generated from brain, liver, muscle and heart tissues of Giant, and annotated 15% repetitive sequences in the genome. Additionally, we resequenced other five B. splendens varieties and detected ~3.4M single-nucleotide variations (SNVs) and 27,305 indels.<br>Conclusions: We provide the first chromosome-level genome for the Siamese fighting fish. The genome will lay a valuable foundation for future research on aggression in B. splendens.<br><br>Keywords: Betta splendens; fish genome; aggression; Hi-C; chromosomal genome assembly; resequencing
**Corresponding Author:**	Xin Liu, Ph.D.<br>BGI<br>CHINA
**Corresponding Author Secondary Information:**	
**Corresponding Author's Institution:**	BGI
**Corresponding Author's Secondary Institution:**	
**First Author:**	Xin Liu, Ph.D.
**First Author Secondary Information:**	
**Order of Authors:**	Xin Liu, Ph.D.<br>Guangyi Fan<br>Judy Chan<br>Kailong Ma

Powered by Editorial Manager® and ProduXion Manager® from Aries Systems Corporation

	Simon Ming-Yuen Lee
	Binrui Yang
	He Zhang
	Xianwei Yang
	Chengcheng Shi
	Henry Law
	Zhitao Ren
	Qiwu Xu
	Qun Liu
	Jiahao Wang
	Wenbin Chen
	Libin Shao
	David Gonçalves
	Andreia Ramos
	Sara Cardoso
	Min Guo
	Jing Cai
	Xun Xu
	Jian Wang
	Huanming Yang
	Yitao Wang
**Order of Authors Secondary Information:**	
**Opposed Reviewers:**	
**Additional Information:**	
**Question**	**Response**
Are you submitting this manuscript to a special series or article collection?	No
**Experimental design and statistics**<br><br>Full details of the experimental design and statistical methods used should be given in the Methods section, as detailed in our Minimum Standards Reporting Checklist. Information essential to interpreting the data presented should be made available in the figure legends.<br><br>Have you included all the information requested in your manuscript?	Yes
**Resources**<br><br>A description of all resources used,	Yes

including antibodies, cell lines, animals and software tools, with enough information to allow them to be uniquely identified, should be included in the Methods section. Authors are strongly encouraged to cite Research Resource Identifiers (RRIDs) for antibodies, model organisms and tools, where possible.<br><br>Have you included the information requested as detailed in our Minimum Standards Reporting Checklist?	
**Availability of data and materials**<br><br>All datasets and code on which the conclusions of the paper rely must be either included in your submission or deposited in publicly available repositories (where available and ethically appropriate), referencing such data using a unique identifier in the references and in the "Availability of Data and Materials" section of your manuscript.<br><br>Have you have met the above requirement as detailed in our Minimum Standards Reporting Checklist?	Yes

Manuscript
Click here to download Manuscript MS_Manuscript_final.doc

High-quality reference genome of the Siamese fighting fish *Betta splendens*, a model species for the study of aggression

Guangyi Fan[1,2,3,4,*], Judy Chan[1,*], Kailong Ma[3,4,*], Simon Ming-Yuen Lee[1,*,†].Binrui Yang[1], He Zhang[2,3,4], Xianwei Yang[2,3,4], Chengcheng Shi[2,3,4], Henry Law[1], Zhitao Ren[1], Qiwu Xu[2,3,4], Qun Liu[2,3,4], Jiahao Wang[2,3,4], Wenbin Chen[3,4], Libin Shao[2,3,4], David Gonçalves[6], Andreia Ramos[6], Sara D. Cardoso[7], Min Guo[1], Jing Cai[1], Xun Xu[2,3,4], Jian Wang[3,5], Huanming Yang[3,5], Xin Liu[2,3,4,†], Yitao Wang[1,†]

[1]State Key Laboratory of Quality Research in Chinese Medicine, Institute of Chinese Medical Sciences, University of Macau, Macao, China.

[2]BGI-Qingdao, Qingdao 266500, China

[3]BGI-Shenzhen, Shenzhen 518083, China

[4]China National GeneBank, BGI-Shenzhen, Shenzhen 518120, China

[5]James D. Watson Institute of Genome Sciences, Hangzhou 310058, China

[6]Institute of Science and Environment, University of Saint Joseph, Macao SAR, China.

[7]Instituto Gulbenkian de Ciência, Oeiras, Portugal

*These authors contributed equally to this work.

[†]Correspondence authors: Yitao Wang (ytwang@umac.mo), Simon Ming-Yuen Lee (simonlee@umac.mo) and Xin Liu (liuxin@genomics.cn).

Abstract

Background: Siamese fighting fish *Betta splendens* are notorious for their aggressiveness and accordingly have been widely used to study aggression. However, the lack of a reference genome has so far limited the understanding of the genetic basis of aggression in this species. Here we present the first reference genome assembly of the Siamese fighting fish.

Findings: We first sequenced and *de novo* assembled a 465.24 Mb genome for the *B. splendens* variety Giant, with a weighted average (N50) scaffold size of 949.03 Kb and an N50 contig size of 19.01 Kb, covering 99.93% of the estimated genome size. To obtain a chromosome-level genome assembly, we constructed one Hi-C library and

sequenced 75.24 Gb reads using the BGISEQ-500 platform. We anchored approximately 93% of the scaffold sequences into 21 chromosomes and evaluated the quality of our assembly using the high contact frequency heatmap and BUSCO. We also performed comparative chromosome analyses between *Oryzias latipes* and *B. splendens,* revealing a chromosome conservation evolution in *B. splendens*. We predicted a total of 23,202 genes assisted by RNA-seq data generated from brain, liver, muscle and heart tissues of Giant, and annotated 15% repetitive sequences in the genome. Additionally, we resequenced other five *B. splendens* varieties and detected ~3.4M single-nucleotide variations (SNVs) and 27,305 indels.

Conclusions: We provide the first chromosome-level genome for the Siamese fighting fish. The genome will lay a valuable foundation for future research on aggression in *B. splendens*.

Keywords: *Betta splendens;* fish genome; aggression; Hi-C; chromosomal genome assembly; resequencing

Data Description

Males of the Siamese fighting fish *Betta splendens* are notorious for their aggressiveness. In nature, males establish and vigorously defend territories where they construct a bubble nest to hold fertilized eggs. In laboratory settings, males will readily attack an opponent, their mirror image, physical models of conspecifics or video images of other males, and accordingly the species has been widely used to study the neurobiological mechanisms of aggression. However, the lack of a reference genome limited so far studies on the genetic basis of aggression in *B. splendens*. The species is also one of the most relevant for the ornamental fish trade as it is easy to keep and reproduce in captivity and throughout its long domestication period many varieties have been selected for their exuberant fins and colors, size or aggressive behavior. Here, we sequenced the genome of *B. splendens* to provide the genomic foundation for future research on aggression and development of genomic tools.

Sampling and sequencing

We purchased five different varieties of adult male Siamese fighting fish including Giant, Half-moon (HM), Half-moon plakat (HMPK), Fighter, and Elephant Ear (EE) from HK supplier TC Northern Betta (**Supplementary Fig. 1**). We constructed and sequenced five DNA libraries for the *B. splendens* variety Giant, including three short insert size libraries and three mate-pair libraries (**Supplementary Table 1**), and five RNA-seq libraries (**Supplementary Table 2**) using the HiSeq 2000 sequencing platform. One Hi-C library for Giant was also constructed and sequenced using the BGISEQ-500 sequencing platform, yielding 75.24 Gb of reads. Additionally, we sequenced five short insert size DNA libraries for the other five *B. splendens* varieties.

Genome assembly

We obtained 52.34 Gb of clean reads using SOAPnuke[1] with strict parameters, including removal of low-quality reads, adapter contamination and PCR duplicates. Then, we performed the *de novo* assembly of the Giant high-quality reads using the SOAP*denovo*[2] assembler. For the genome assembly, the short insert size libraries were used to construct the contig sequences and the mate-paired libraries were used to link the scaffolds. We

filled the gaps within the scaffolds using Gapcloser. We obtained a genome with a size of 465.24 Mb, with an N50 scaffold size of 949.03 Kb and an N50 contig size of 19.01 Kb (**Table 1**), covering 99.93% of the estimated genome size of 465.55 Mb using kmer analysis (**Supplementary Table 3** and **Supplementary Fig. 2**). To construct the reference genome at the chromosome-level, we used a MBOI endonuclease to cut the DNA, and constructed a Hi-C library based on a previous protocol[3]. We sequenced 75.24 Gb of data using the BGISEQ-500 sequencing platform, and obtained 34.5Gb valid reads (~45.8%) that could be used to anchor the scaffolds into chromosomes after quality control using the HiC-Pro pipeline[4] (**Supplementary Fig. 3-7**). Lastly, we constructed 21 chromosomes that occupied 95.3% of the genome (**Fig. 1**, **Table 1** and **Supplementary Table 4**) using Juicer[5] and 3D-dna pipeline[6] based on the draft genome assembly. To evaluate the quality of the assembly, we found 95.4% of BUSCO genes that could be completely covered by our genome (**Table 2**) and approximately 98% of the transcripts assembled from four RNA-seq data could be aligned against the genome with more than 90% coverage, revealing the high quality of the assembly (**Supplementary Table 5**).

Genome annotation

We annotated the repetitive sequences by combining *de novo*- and homolog-based approaches. We firstly used LTR-FINDER[7] and RepeatModeler to construct a repetitive sequence library, and then used RepeatMasker[8] to classify these repeat sequences. We also detected repetitive sequences using RepeatMasker and ProteinMasker based on the Repbase library[9]. We identified a total of 15.12% transposable elements in the genome (**Supplementary Table 6**).

For the protein-coding prediction we combined several approaches: 1) gene model prediction using Augustus[10] and GENSCAN[11]; 2) gene prediction using GeneWise[12] based on the alignment results of protein sequences of other published species against our assembly; and 3) four RNA-seq data samples were used to assist in predicting the gene structure with Cufflinks[13]. Lastly, we integrated all of this evidence into a non-redundancy gene set using GLEAN[14]. The final gene set contained 23,202 genes (**Supplementary Table 7**), close to the number for *Oryzias latipes*[15] (24,674) and slightly less than that for *Danio rerio*[16] (26,046). We identified 90% of the 2,586 BUSCO gene

models to be complete in the gene set (**Table 2**), indicating the high-quality assembly and annotation.

Comparative genomic analysis

We compared the fighting fish genome with other species, both at the whole genome- and gene-level. All of the 21 chromosomes assembled for the fighting fish could be matched to chromosomes of *Oryzias latipes* with a mean coverage ratio of 75.3%. From these, 18 chromosomes had a single hit to one chromosome of *O. latipes*, and 3 chromosomes (1, 19 and 21) had a hit in two chromosomes of *O. latipes* (**Fig. 2** and **Supplementary Table 8**), indicating conservative evolution for most of chromosomes, as well as several chromosome reshuffling events between these two species. Furthermore, from the gene set level, KO (KEGG Orthology) terms of animals from 109 different species were counted and compared with the fighting fish gene set using the KEGG database[17] (version 79). There were five KO terms notably expanded in fighting fish compared with all other animals, including 147 NACHT, LRR and PYD domains-containing protein 3 (*NLRP3*, K12800), 86 tripartite motif-containing protein 47 (*TRIM47*, K12023), 43 chloride channel 7 (*CLCN7*, K05016), 29 arginine vasopressin receptor 2 (*AVPR2*, K04228) and 17 maltase-glucoamylase (*MGAM*, K12047) (**Fig. 3**). *NLRP3* has two prominent expansions, corresponding to clade 1, containing 56 genes, and clade2, containing 79 genes, whereas other fish species in these two clades have less than three gene copies (**Fig. 4**). *NLRP3* encodes a pyrin-like protein containing a pyrin domain, a nucleotide-binding site (NBS) domain, and a leucine-rich repeat (LRR) motif, and plays a role in the regulation of inflammation, the immune response, and apoptosis[18].

Resequencing

To evaluate the genetic diversity among the four varieties of *Betta splendens*, we called the SNVs (single-nucleotide variations) and Indels (insertions and deletions) based on the read alignment result using Giant assembly as a reference. We obtained 70.25 Gb of clean reads filtered from 79.18 Gb of raw reads (**Supplementary Table 9**). We used BWA[19] (v0.6.2) to align all the re-sequencing data to the reference genome and the UnifiedGenotyper in Genome Analysis Toolkit (GATK[20], v2.8.1) to call variations. In

total, we detected approximately 3.4 M SNVs and 27,305 indels, which will provide abundant genetic polymorphism for use in future research and applications. Moreover, we found that the cluster result was in accordance with the behavior shown by each *B. splendens* variety, by constructing a phylogenetic tree and performing a PCA analysis (**Supplementary Fig. 8**).

Availability of data

We have deposited the data into the GenBank under the BioProject number PRJNA416843.

Abbreviations

bp: base pair; Gb: gigabases; Kb: kilobases; KO: KEGG Orthology; M: million; Mb: megabases; PCA: principal component analysis; SNV: single-nucleotide variation

Acknowledgements

We thank the Macau Science and Technology Development Fund for financial support (project 011/2014/A1), and Shenzhen Municipal Government of China(JCYJ20151015162041454 to W.C. and JCYJ20150529150505656 to X.L.).

Authors contributions

G.F., S.L. and J.C. conceived the project. G.F., X.L. and J.C. supervised the research. H.Z., C.S. and X.Y. conceived and designed the experiments. K.M., X.L. and B.Y. performed genome assembly and gene annotation. H.L., Z.R., Q.L. and Q.X. prepared the fighting fish sample. Jiahao.W., W.C., X.X. and L.S. performed sequencing. A.R., M.G., Jing.C., H.Y. and J.W. performed comparative genomic analysis. G.F., S.C., Y. W. and D.G. revised the paper.K.M. and X.Y. performed data accession.

Tables

Table 1. Statistics of the assembly using SOAP*denovo* and Hi-C data.

Type	Scaffold Original	Contig Original	Scaffold (Hi-C)	Contig (Hi-C)
Total Number	92,886	138,929	3,057	48,943
Toltal length (bp)	465,240,853	421,527,246	452,537,980	409,013,355
Average length (bp)	5008.73	3034.12	19,754,490	19,674
N50 Length (bp)	949,032	19,014	35,205,731	189,132
N90 Length (bp)	59,769	3,504	14,840,089	4,434

Table 2. Evaluation results of the genome and gene set using BUSCO.

	Genome		Genes	
	Number	Percentage (%)	Number	Percentage (%)
Complete	2,466	95.4	2,327	90.0
Single-copy complete	2,422	93.7	2,254	87.2
Duplicated complete	44	1.7	73	2.8
Fragmented	69	2.7	204	7.9
Missing	51	1.9	55	2.1
Total	2,586	-	2,586	-

Figure legends

Fig. 1. Hi-C interaction heatmap for *B. Splendens* reference genome, showing interactions between the 21 chromosomes.

Fig. 2. Collinear relationship between *B. splendens* and *Oryzias latipes*. Green represents the chromosomes of *B. splendens* and the other multicolor represent the chromosomes of *O. latipes*.

Fig. 3. Five gene families with prominent expansion in *B. splendens* when compared with other species.

Fig. 4. The gene phylogenetic tree of NLRP3 gene family (KO: K12800) using the genes of *B. splendens* and other species. Clade 1 and clade 2 show two prominent expansion sub-families of *B. splendens*.

References

1. Chen, Y. *et al.* SOAPnuke: a MapReduce acceleration-supported software for integrated quality control and preprocessing of high-throughput sequencing data. *GigaScience* **7**, 1-6 (2018).
2. Luo, R. *et al.* SOAPdenovo2: an empirically improved memory-efficient short-read de novo assembler. *Gigascience* **1**, 18 (2012).
3. Durand, N.C. *et al.* Juicer Provides a One-Click System for Analyzing Loop-Resolution Hi-C Experiments. *Cell Syst* **3**, 95-8 (2016).
4. Servant, N. *et al.* HiC-Pro: an optimized and flexible pipeline for Hi-C data processing. *Genome Biol* **16**, 259 (2015).
5. Belton, J.M. *et al.* Hi-C: a comprehensive technique to capture the conformation of genomes. *Methods* **58**, 268-76 (2012).
6. Dudchenko, O. *et al.* De novo assembly of the Aedes aegypti genome using Hi-C yields chromosome-length scaffolds. *Science* **356**, 92-95 (2017).
7. Xu, Z. & Wang, H. LTR_FINDER: an efficient tool for the prediction of full-length LTR retrotransposons. *Nucleic Acids Res* **35**, W265-8 (2007).
8. Tarailo-Graovac, M. & Chen, N. Using RepeatMasker to identify repetitive elements in genomic sequences. *Curr Protoc Bioinformatics* **Chapter 4**, Unit 4 10 (2009).
9. Jurka, J. *et al.* Repbase Update, a database of eukaryotic repetitive elements. *Cytogenet Genome Res* **110**, 462-7 (2005).
10. Stanke, M. *et al.* AUGUSTUS: ab initio prediction of alternative transcripts. *Nucleic Acids Res* **34**, W435-9 (2006).
11. Burge, C. & Karlin, S. Prediction of complete gene structures in human genomic DNA. *J Mol Biol* **268**, 78-94 (1997).
12. Birney, E. GeneWise and Genomewise. *Genome Research* **14**, 988-995 (2004).
13. Trapnell, C. *et al.* Differential gene and transcript expression analysis of RNA-seq experiments with TopHat and Cufflinks. *Nature Protocols* **7**, 562-578 (2012).
14. Elsik, C.G. *et al.* Creating a honey bee consensus gene set. *Genome Biol* **8**, R13 (2007).
15. Kasahara, M. *et al.* The medaka draft genome and insights into vertebrate genome evolution. *Nature* **447**, 714-719 (2007).
16. Howe, K. *et al.* The zebrafish reference genome sequence and its relationship to the human genome. *Nature* **496**, 498-503 (2013).
17. Kanehisa, M. The KEGG Database. **247**, 91-103 (2002).
18. Hirota, S.A. *et al.* NLRP3 inflammasome plays a key role in the regulation of intestinal homeostasis. *Inflammatory Bowel Diseases* **17**, 1359-1372 (2011).

19. Li, H. & Durbin, R. Fast and accurate short read alignment with Burrows-Wheeler transform. *Bioinformatics* **25**, 1754-1760 (2009).

20. McKenna, A. *et al.* The Genome Analysis Toolkit: A MapReduce framework for analyzing next-generation DNA sequencing data. *Genome Research* **20**, 1297-1303 (2010).

Fig.1

Click here to download Figure Fig.1.pdf

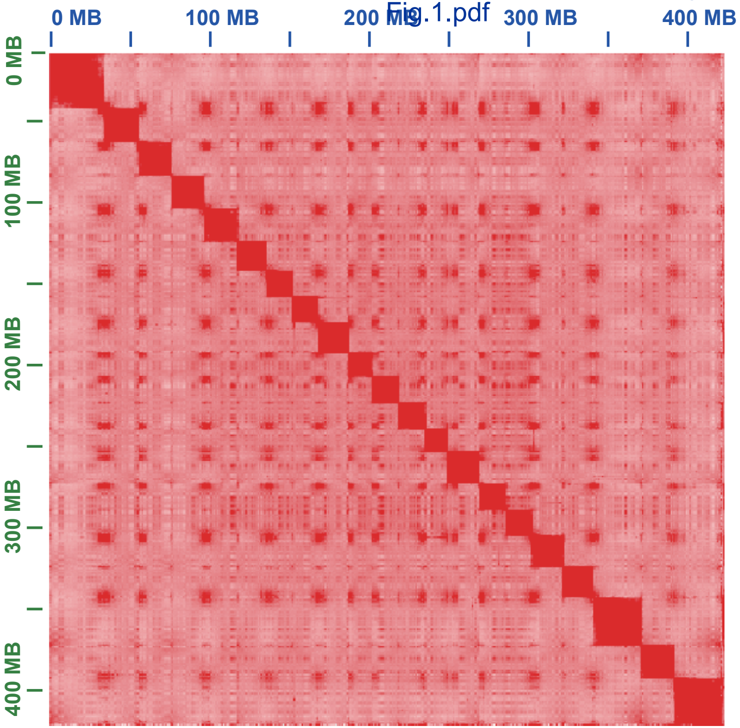

Fig.2

Click here to download Figure Fig.2.png

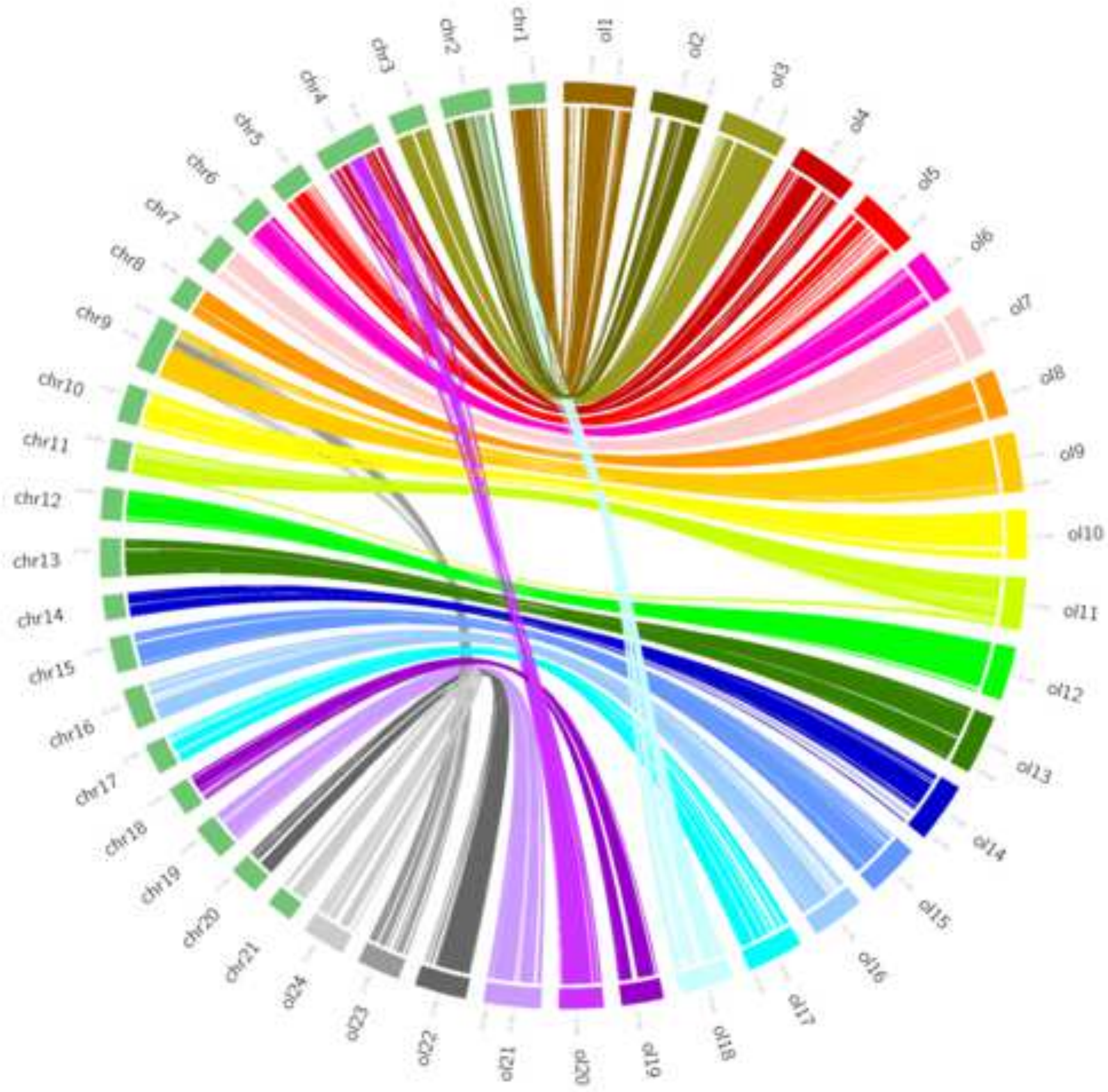

Fig.3

Click here to download Figure

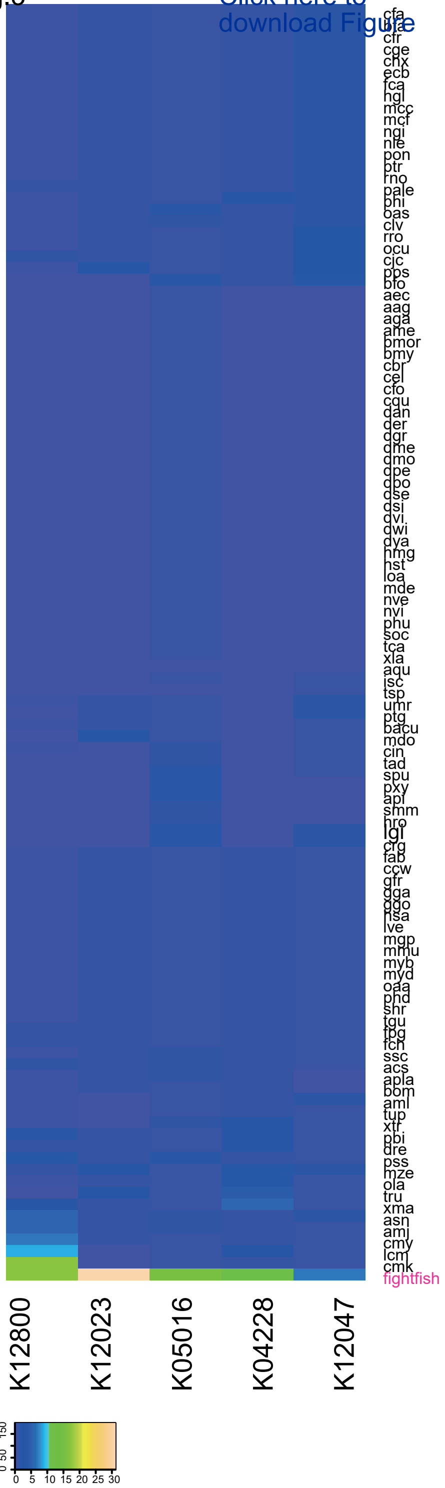

Fig.4

Click here to download Figure Fig.4.pdf

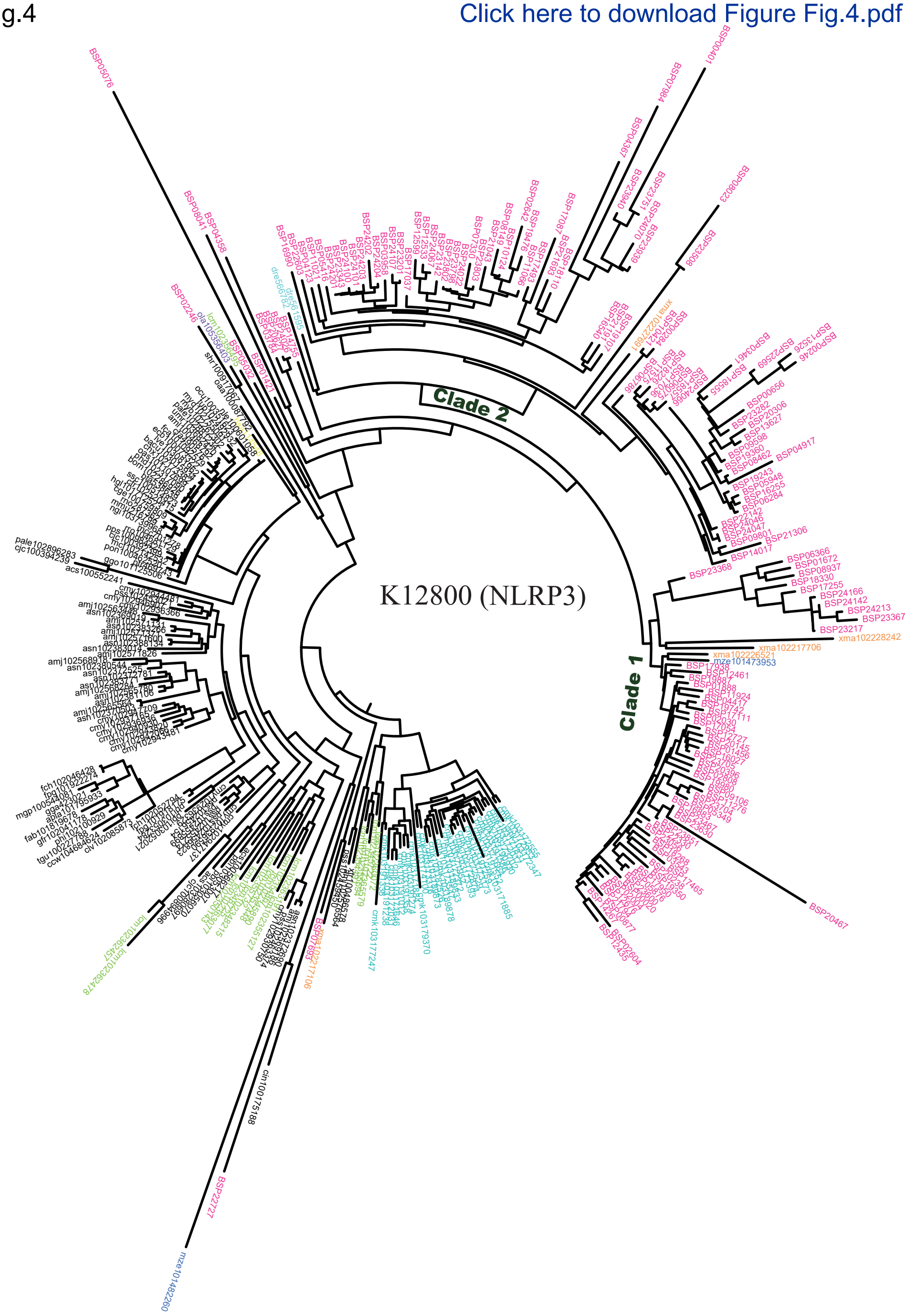

Click here to access/download
Supplementary Material
Supplementary material.docx
