# Supplementary material for: Chromosome-level reference genome of the Siamese fighting fish Betta splendens, a model species for the study of aggression
Source: Gigascience. 2018 Jul 11;7(11):giy087. doi: 10.1093/gigascience/giy087 (PMC6251983; doi:10.1093/gigascience/giy087)
Supplement: GIGA-D-18-00046_Revision_2.pdf [file giy087_giga-d-18-00046_revision_2.pdf]

GigaScience

Chromosome-level reference genome of the Siamese fighting fish Betta splendens, a model species for the study of aggression

--Manuscript Draft--

**Manuscript Number:**	GIGA-D-18-00046R2
**Full Title:**	Chromosome-level reference genome of the Siamese fighting fish Betta splendens, a model species for the study of aggression
**Article Type:**	Data Note
**Funding Information:**	Macau Science and Technology Development Fund (011/2014/A1) — dr. Guangyi Fan<br>Shenzhen Municipal Government of China (JCYJ20151015162041454) — dr. Wenbin Chen<br>Shenzhen Municipal Government of China (JCYJ20150529150505656) — Dr. Xin Liu
**Abstract:**	Background: Siamese fighting fish Betta splendens (NCBI Taxonomy ID: 158456) are notorious for their aggressiveness and accordingly have been widely used to study aggression. However, the lack of a reference genome has so far limited the understanding of the genetic basis of aggression in this species. Here we present the first reference genome assembly of the Siamese fighting fish.<br>Findings: We first sequenced and de novo assembled a 465.24 Mb genome for the B. splendens variety Giant, with a weighted average (N50) scaffold size of 949.03 Kb and an N50 contig size of 19.01 Kb, covering 99.93% of the estimated genome size. To obtain a chromosome-level genome assembly, we constructed one Hi-C library and sequenced 69.7 Gb reads using the BGISEQ-500 platform. We anchored approximately 93% of the scaffold sequences into 21 chromosomes and evaluated the quality of our assembly using the high contact frequency heatmap and BUSCO. We also performed comparative chromosome analyses between Oryzias latipes and B. splendens, revealing a chromosome conservation evolution in B. splendens. We predicted a total of 23,981 genes assisted by RNA-seq data generated from brain, liver, muscle and heart tissues of Giant, and annotated 15% repetitive sequences in the genome. Additionally, we resequenced other five B. splendens varieties and detected ~3.4M single-nucleotide variations (SNVs) and 27,305 indels.<br>Conclusions: We provide the first chromosome-level genome for the Siamese fighting fish. The genome will lay a valuable foundation for future research on aggression in B. splendens.<br><br>Keywords: Betta splendens; fish genome; aggression; Hi-C; chromosomal genome assembly; resequencing
**Corresponding Author:**	Xin Liu, Ph.D.<br>BGI<br>CHINA
**Corresponding Author Secondary Information:**	
**Corresponding Author's Institution:**	BGI
**Corresponding Author's Secondary Institution:**	
**First Author:**	Xin Liu, Ph.D.
**First Author Secondary Information:**	
**Order of Authors:**	Xin Liu, Ph.D.<br>Guangyi Fan<br>Judy Chan<br>Kailong Ma

Powered by Editorial Manager® and ProduXion Manager® from Aries Systems Corporation

	Binrui Yang
	He Zhang
	Xianwei Yang
	Chengcheng Shi
	Henry Law
	Zhitao Ren
	Qiwu Xu
	Qun Liu
	Jiahao Wang
	Wenbin Chen
	Libin Shao
	David Gonçalves
	Andreia Ramos
	Sara Cardoso
	Min Guo
	Jing Cai
	Xun Xu
	Jian Wang
	Huanming Yang
	Yitao Wang
**Order of Authors Secondary Information:**	
**Response to Reviewers:**	Dear edit,<br><br>We are very grateful to you and the reviewers for re-review our submission.We are honored that our manuscript can be accepted for publication in GigaScience.We made changes based on your suggestions and responded point-by-point.<br><br>1. Please include the version numbers for all software tools you used.<br>Answer: We have added the version number of the software tool.<br><br>2. Thank you for using protocols.io, to share protocols, that's great. Please include the protocols in your bibliography and cite them in your text where appropriate. For example, when referring to the DNA extraction method in the manuscript, you should cite your respective protocol: [xx] Ma K. DNA extraction for the Betta splendens genome. Protocols.io. 2018. http://dx.doi.org/10.17504/protocols.io.qvedw3e ... and the same way for your other protocols (please note that each protocol has an individual doi number).<br>Answer: We have added the protocol to the bibliography and cite them in my text where appropriate.<br><br>3. Please mention the fish base ID for the species in the manuscript<br>Answer: We added the NCBI Taxonomy ID to identify this species.<br><br>4. I included a citation to your GigaDB dataset in the data availability section, please double check.<br>Answer: Thank you for your help in this section. We have updated a citation to my GigaDb dataset at the bottom of the text.<br><br>5. I resolved some "tracked changes" in the attached version of the manuscript, please double check this is now the correct version.<br>Answer: Thank you for helping us solve the "tracked changes". We have checked it

	again.<br><br>6.Please check your supplementary material - I think there are still some "tracked changes" that need to be resolved.<br>Answer: We checked and solved the "tracked changes" in the supplementary material<br><br>7. I included some Research Resource Identifiers (RRIDs) for software tools you used - please double check.<br>Answer: Thank you for your help in this section.I checked the Research Resource Identifiers you added and added the RRID of the other software tools.
**Additional Information:**	
**Question**	**Response**
Are you submitting this manuscript to a special series or article collection?	No
**Experimental design and statistics**<br><br>Full details of the experimental design and statistical methods used should be given in the Methods section, as detailed in our Minimum Standards Reporting Checklist. Information essential to interpreting the data presented should be made available in the figure legends.<br><br>Have you included all the information requested in your manuscript?	Yes
**Resources**<br><br>A description of all resources used, including antibodies, cell lines, animals and software tools, with enough information to allow them to be uniquely identified, should be included in the Methods section. Authors are strongly encouraged to cite Research Resource Identifiers (RRIDs) for antibodies, model organisms and tools, where possible.<br><br>Have you included the information requested as detailed in our Minimum Standards Reporting Checklist?	Yes
**Availability of data and materials**<br><br>All datasets and code on which the	Yes

Powered by Editorial Manager® and ProduXion Manager® from Aries Systems Corporation

conclusions of the paper rely must be either included in your submission or deposited in publicly available repositories (where available and ethically appropriate), referencing such data using a unique identifier in the references and in the “Availability of Data and Materials” section of your manuscript.<br><br>Have you have met the above requirement as detailed in our Minimum Standards Reporting Checklist?	

Manuscript

Click here to access/download;Manuscript;MS_FightingFish_0807.doc

Click here to view linked References

Chromosome-level reference genome of the Siamese fighting fish *Betta splendens*, a model species for the study of aggression

Guangyi Fan[1,2,3,4,*], Judy Chan[1,*], Kailong Ma[3,4,*], Binrui Yang[1], He Zhang[2,3,4], Xianwei Yang[2,3,4], Chengcheng Shi[2,3,4], Henry Law[1], Zhitao Ren[1], Qiwu Xu[2,3,4], Qun Liu[2,3,4], Jiahao Wang[2,3,4], Wenbin Chen[3,4], Libin Shao[2,3,4], David Gonçalves[6], Andreia Ramos[6], Sara D. Cardoso[7], Min Guo[1], Jing Cai[1], Xun Xu[2,3,4], Jian Wang[3,5], Huanming Yang[3,5], Xin Liu[2,3,4,†], Yitao Wang[1,†]

[1]State Key Laboratory of Quality Research in Chinese Medicine, Institute of Chinese Medical Sciences, University of Macau, Macao, China.

[2]BGI-Qingdao, BGI-Shenzhen, Qingdao, 266555, China

[3]BGI-Shenzhen, Shenzhen 518083, China

[4]China National GeneBank, BGI-Shenzhen, Shenzhen 518120, China

[5]James D. Watson Institute of Genome Sciences, Hangzhou 310058, China

[6]Institute of Science and Environment, University of Saint Joseph, Macao SAR, China.

[7]Instituto Gulbenkian de Ciência, Oeiras, Portugal

*These authors contributed equally to this work.

[†]Correspondence authors: Yitao Wang (ytwang@umac.mo) and Xin Liu (liuxin@genomics.cn).

Abstract

Background: Siamese fighting fish *Betta splendens* (NCBI Taxonomy ID: 158456) are notorious for their aggressiveness and accordingly have been widely used to study aggression. However, the lack of a reference genome has so far limited the understanding of the genetic basis of aggression in this species. Here we present the first reference genome assembly of the Siamese fighting fish.

Findings: We first sequenced and *de novo* assembled a 465.24 Mb genome for the *B. splendens* variety Giant, with a weighted average (N50) scaffold size of 949.03 Kb and an N50 contig size of 19.01 Kb, covering 99.93% of the estimated genome size. To obtain a chromosome-level genome assembly, we constructed one Hi-C library and

sequenced 75.24 Gb reads using the BGISEQ-500 platform. We anchored approximately 93% of the scaffold sequences into 21 chromosomes and evaluated the quality of our assembly using the high contact frequency heatmap and BUSCO. We also performed comparative chromosome analyses between *Oryzias latipes* and *B. splendens,* revealing a chromosome conservation evolution in *B. splendens*. We predicted a total of 23,981 genes assisted by RNA-seq data generated from brain, liver, muscle and heart tissues of Giant, and annotated 15% repetitive sequences in the genome. Additionally, we resequenced other five *B. splendens* varieties and detected ~3.4M single-nucleotide variations (SNVs) and 27,305 indels.

Conclusions: We provide the first chromosome-level genome for the Siamese fighting fish. The genome will lay a valuable foundation for future research on aggression in *B. splendens*.

Keywords: *Betta splendens;* fish genome; aggression; Hi-C; chromosomal genome assembly; resequencing

Data Description

Males of the Siamese fighting fish *Betta splendens* are notorious for their aggressiveness. In nature, males establish and vigorously defend territories where they construct a bubble nest to hold fertilized eggs. In laboratory settings, males will readily attack an opponent, their mirror image, physical models of conspecifics or video images of other males, and accordingly the species has been widely used to study the neurobiological mechanisms of aggression. However, the lack of a reference genome limited so far studies on the genetic basis of aggression in *B. splendens*. The species is also one of the most relevant for the ornamental fish trade as it is easy to keep and reproduce in captivity and throughout its long domestication period many varieties have been selected for their exuberant fins and colors, size or aggressive behavior. Here, we sequenced the genome of *B. splendens* to provide the genomic foundation for future research on aggression and development of genomic tools.

Sampling and sequencing

We purchased five different varieties of adult male Siamese fighting fish including Giant, Half-moon (HM), Half-moon plakat (HMPK), Fighter, and Elephant Ear (EE) from Hong Kong supplier TC Northern Betta for DNA and RNA extraction[1,2] (**Supplementary Fig. 1**). We constructed and sequenced six DNA libraries for the *B. splendens* variety Giant, including three short insert size libraries and three mate-pair libraries (**Supplementary Table 1**), and five RNA-seq libraries (**Supplementary Table 2**) using the HiSeq 2000 sequencing platform. One Hi-C library for Giant was also constructed and sequenced using the BGISEQ-500 sequencing platform, yielding 75.24 Gb of reads. Additionally, we sequenced four short insert size DNA libraries for the other four *B. splendens* varieties.

Genome assembly

We obtained 52.34 Gb of clean reads using SOAPnuke, version 1.5.3 (SOAPnuke, RRID:SCR_015025)[3], with strict parameters, including removal of low-quality reads, adapter contamination and PCR duplicates. Then, we performed the *de novo* assembly of the Giant reads using the SOAPdenovo2, version 2.04 (SOAPdenovo2,

RRID:SCR_014986)[4], assembler. For the genome assembly, the short insert size libraries were used to construct the contig sequences and the mate-paired libraries were used to link the scaffolds. We filled the gaps within the scaffolds using GapCloser, version 1.12 (GapCloser, RRID:SCR_015026). We obtained a genome assembly with a size of 465.24 Mb, with an N50 scaffold size of 949.03 Kb and an N50 contig size of 19.01 Kb (**Table 1**), covering 99.93% of the estimated genome size of 465.55 Mb using kmer, version 1.0, analysis (**Supplementary Table 3** and **Supplementary Fig. 2**). To construct the reference genome at the chromosome-level, we used a MBOI endonuclease to cut the DNA, and constructed a Hi-C library based on a previous protocol[5]. We sequenced 75.24 Gb of data using the BGISEQ-500 sequencing platform, and obtained 34.5Gb valid reads (~45.8%) that could be used to anchor the scaffolds into chromosomes after quality control using the HiC-Pro, version 2.8.0, pipeline[6,7] (**Supplementary Fig. 3-7**). Lastly, we constructed 21 chromosomes that occupied 95.3% of the genome (**Fig. 1**, **Table 1** and **Supplementary Table 4**) using Juicer[8], version 1.5, and 3D-dna, version 170123, pipeline[9] based on the draft genome assembly. To evaluate the quality of the assembly, we found 95.4% of BUSCO version 3.0.1 (BUSCO, RRID:SCR 015008) genes that could be completely covered by our genome (**Table 2**) and approximately 98% of the transcripts assembled from RNA-seq data could be aligned against the genome with more than 90% coverage (**Supplementary Table 5**).

Genome annotation

We annotated the repetitive sequences by combining *de novo* and homolog-based approaches[10]. We firstly used LTR-FINDER, version 1.06 (LTR_Finder, RRID:SCR_015247)[11], and RepeatModeler, version 1.0.8 (RepeatModeler, RRID:SCR_015027), to construct a repetitive sequence library, and then used RepeatMasker, version 3.3.0 (RepeatMasker, RRID:SCR_012954)[12], to classify these repeat sequences. We also detected repetitive sequences using RepeatMasker and ProteinMasker, version 3.3.0, based on the Repbase library[13]. We identified a total of 15.12% transposable elements in the genome (**Supplementary Table 6**).

For the protein-coding prediction we combined several approaches: 1) gene model prediction using AUGUSTUS, version 3.0.3 (Augustus, RRID:SCR_008417)[14], and

GENSCAN, version 1.0 (GENSCAN, RRID:SCR_012902)[15]; 2) gene prediction using GeneWise, version 2.2.0 (GeneWise, RRID:SCR_015054)[16], based on the alignment results of protein sequences of other published species against our assembly; and 3) five RNA-seq libraries were used to assist in predicting the gene structure with Cufflinks, version 2.2.1 (Cufflinks, RRID:SCR_014597)[17]. Lastly, we integrated all of this evidence into a non-redundancy gene set using GLEAN[18], version 1.0. The final gene set contained 23,981 genes (**Supplementary Table 7**), close to the number for *Oryzias latipes*[19] (24,674) and slightly less than that for *Danio rerio*[20] (26,046). We identified 90% of the 4,128 BUSCO gene models to be complete in the actinopterygii gene set (**Table 2**).

Comparative genomic analysis

We compared the fighting fish genome with other species using Lastz , version 1.02.00, both at the whole genome- and gene-level. All of the 21 chromosomes assembled for the fighting fish could be matched to chromosomes of *Oryzias latipes* with a mean coverage ratio of 75.3%. From these, 18 chromosomes had a single hit to one chromosome of *O. latipes,* and 3 chromosomes (1, 19 and 21) had a hit in two chromosomes of *O. latipes* (**Fig. 2** and **Supplementary Table 8**), indicating conservative evolution for most of chromosomes, as well as several chromosome reshuffling events between these two species. Furthermore, from the gene set level, KO (KEGG Orthology) terms of animals from 109 different species were counted and compared with the fighting fish gene set using the KEGG database[21] , version 79. There were five KO terms notably expanded in fighting fish compared with all other animals, including 147 NACHT, LRR and PYD domains-containing protein 3 (*NLRP3*, K12800), 86 tripartite motif-containing protein 47 (*TRIM47*, K12023), 43 chloride channel 7 (*CLCN7*, K05016), 29 arginine vasopressin receptor 2 (*AVPR2*, K04228) and 17 maltase-glucoamylase (*MGAM*, K12047) (**Fig. 3**). *NLRP3* has two prominent expansions, corresponding to clade 1, containing 56 genes, and clade2, containing 79 genes, whereas other fish species in these two clades have less than three gene copies (**Fig. 4**). *NLRP3* encodes a pyrin-like protein containing a pyrin domain, a nucleotide-binding site (NBS) domain, and a leucine-rich repeat (LRR) motif, and plays a role in the regulation of inflammation, the immune response, and apoptosis[22].

Resequencing

We found through Mirror-Image Stimulation (MIS) test that the different varieties of the Siamese fighting are different in aggressiveness. Males of *B. splendens* were tested under a standardized mirror-elicited aggression paradigm as this elicits similar aggression levels to those of a real conspecific. One fighting fish was located into the testing tank (30 x 19 x 23 cm) and left undisturbed for 30 min for acclimation. Then, the swimming behavior was recorded by taking 5-min video by a side digital camera and the swimming track was recorded by Viewpoint ZebraLab Tracking System for 5 min. This represented the control state. After that, a mirror of similar size with the side wall was placed into the tank to induce aggression of the fish by its own mirror image. Aggression of fighting fish was observed through the following behaviors: opecular flare, fin spreading, 90º turn and mirror hit. As expected, the mirror image elicited a high frequency of aggressive displays. Fish spent most time close to the mirror side and increased overall swimming distance as compared to controls. Within the all tested varieties, Giant had overall the highest frequency of aggressive displays and HM the lowest (**Supplementary Fig.8**).

To evaluate the genetic diversity among the four varieties of *Betta splendens*, we called the SNVs (single-nucleotide variations) and Indels (insertions and deletions) based on the read alignment result using Giant assembly as a reference. We obtained 70.25 Gb of clean reads filtered from 79.18 Gb of raw reads (**Supplementary Table 9**). We used BWA, version0.6.2 (BWA, RRID:SCR_010910)[23], to align all the re-sequencing data to the reference genome and the UnifiedGenotyper in Genome Analysis Toolkit , version2.8.1 (GATK , RRID:SCR_001876)[24], to call variations. In total, we detected approximately 3.4 M SNVs and 27,305 indels, which will provide abundant genetic polymorphism for use in future research and applications.

Availability of data

We have deposited the data into GenBank under the BioProject accession number PRJNA416843. Supporting data is also available via the GigaScience database GigaDB[25].

Abbreviations

bp: base pair; Gb: gigabases; Kb: kilobases; KO: KEGG Orthology; M: million; Mb: megabases; PCA: principal component analysis; SNV: single-nucleotide variation

Acknowledgements

We thank the Strategic Priority Research Program of the Chinese Academy of Sciences (XDA16010100), the Macau Science and Technology Development Fund for financial support (project 011/2014/A1) and Shenzhen Municipal Government of China(JCYJ20151015162041454 to W.C. and JCYJ20150529150505656 to X.L.)

Authors contributions

G.F., S.L. and J.C. conceived the project. G.F., X.L. and J.C. supervised the research. H.Z., C.S. and X.Y. conceived and designed the experiments. K.M., X.L. and B.Y. performed genome assembly and gene annotation. H.L., Z.R., Q.L. and Q.X. prepared the fighting fish sample. Jiahao. W., W.C., X.X. and L.S. performed sequencing. A.R., M.G., Jing. C., H.Y. and J.W. performed comparative genomic analysis. G.F., S.C., Y. W. and D.G. revised the paper. K.M. and X.Y. performed data accession.

Tables

Table 1. Statistics of the assembly using SOAP*denovo* and Hi-C data.

**Type**	**Scaffold Original**	**Contig Original**	**Scaffold (Hi-C)**	**Contig (Hi-C)**
Total Number	92,886	138,929	91,819	139,323
Toltal length (bp)	465,240,853	421,527,246	465,132,837	421,527,246
Average length (bp)	5008.73	3034.12	5,066	3,026
N50 (bp)	949,032	19,014	19,754,490	18,890
N90 (bp)	59,769	3,504	13,781,534	3,470

Table 2. Evaluation results of the genome and gene set using BUSCO.

	Genome		Genes	
	Number	Percentage (%)	Number	Percentage (%)
Complete	4,375	95.4	4,128	90.1
Single-copy complete	4,232	92.3	3,937	85.9
Duplicated complete	142	3.1	191	4.2
Fragmented	128	2.8	338	7.4
Missing	82	1.8	118	2.5
Total	4,584	-	4,584	-

Figure legends

Fig. 1. Hi-C interaction heatmap for *B. Splendens* reference genome, showing interactions between the 21 chromosomes.

Fig. 2. Collinear relationship between *B. splendens* and *Oryzias latipes*. Green represents the chromosomes of *B. splendens* and the other multicolor represent the chromosomes of *O. latipes*.

Fig. 3. Five gene families with prominent expansion in *B. splendens* when compared with other 109 species including *NLRP3* (K12800), *TRIM47* (K12023), *CLCN7* (K05016), *AVPR2* (K04228), *MGAM* (K12047) using the KEGG database.

Fig. 4. The gene phylogenetic tree of NLRP3 gene family (KO: K12800) using the genes of *B. splendens* and other species. Clade 1 and clade 2 show two prominent expansion sub-families of *B. splendens*.

References

1. Ma, K. DNA extraction for the Betta splendens genome. *Protocols.io.* (2018). http://dx.doi.org/10.17504/protocols.io.qvedw3e

2. Ma, K. RNA extraction for the Betta splendens genome. *Protocols.io.* (2018). http://dx.doi.org/10.17504/protocols.io.qvfdw3n
3. Chen, Y. *et al.* SOAPnuke: a MapReduce acceleration-supported software for integrated quality control and preprocessing of high-throughput sequencing data. *GigaScience* **7**, 1-6 (2018).
4. Luo, R. *et al.* SOAPdenovo2: an empirically improved memory-efficient short-read de novo assembler. *GigaScience* **1**(2012).
5. Durand, N.C. *et al.* Juicer Provides a One-Click System for Analyzing Loop-Resolution Hi-C Experiments. *Cell Syst* **3**, 95-8 (2016).
6. Servant, N. *et al.* HiC-Pro: an optimized and flexible pipeline for Hi-C data processing. *Genome Biol* **16**, 259 (2015).
7. Liu, X. The pipeline of Hi-C assembly. *Protocols.io.* (2018). http://dx.doi.org/10.17504/protocols.io.qradv2e
8. Belton, J.M. *et al.* Hi-C: a comprehensive technique to capture the conformation of genomes. *Methods* **58**, 268-76 (2012).
9. Dudchenko, O. *et al.* De novo assembly of the Aedes aegypti genome using Hi-C yields chromosome-length scaffolds. *Science* **356**, 92-95 (2017).
10. Liu, X. An analytical pipeline of assembly and annotation of the Betta splendens genome. *Protocols.io.* (2018). http://dx.doi.org/10.17504/protocols.io.qq9dvz6
11. Xu, Z. & Wang, H. LTR_FINDER: an efficient tool for the prediction of full-length LTR retrotransposons. *Nucleic Acids Res* **35**, W265-8 (2007).
12. Tarailo-Graovac, M. & Chen, N. Using RepeatMasker to identify repetitive elements in genomic sequences. *Curr Protoc Bioinformatics* **Chapter 4**, Unit 4 10 (2009).
13. Jurka, J. *et al.* Repbase Update, a database of eukaryotic repetitive elements. *Cytogenet Genome Res* **110**, 462-7 (2005).
14. Stanke, M. *et al.* AUGUSTUS: ab initio prediction of alternative transcripts. *Nucleic Acids Res* **34**, W435-9 (2006).
15. Burge, C. & Karlin, S. Prediction of complete gene structures in human genomic DNA. *J Mol Biol* **268**, 78-94 (1997).
16. Birney, E. GeneWise and Genomewise. *Genome Research* **14**, 988-995 (2004).
17. Trapnell, C. *et al.* Differential gene and transcript expression analysis of RNA-seq experiments with TopHat and Cufflinks. *Nature Protocols* **7**, 562-578 (2012).
18. Elsik, C.G. *et al.* Creating a honey bee consensus gene set. *Genome Biol* **8**, R13 (2007).
19. Kasahara, M. *et al.* The medaka draft genome and insights into vertebrate genome evolution. *Nature* **447**, 714-719 (2007).
20. Howe, K. *et al.* The zebrafish reference genome sequence and its relationship to the human genome. *Nature* **496**, 498-503 (2013).
21. Kanehisa, M. The KEGG Database. **247**, 91-103 (2002).
22. Hirota, S.A. *et al.* NLRP3 inflammasome plays a key role in the regulation of intestinal homeostasis. *Inflammatory Bowel Diseases* **17**, 1359-1372 (2011).
23. Li, H. & Durbin, R. Fast and accurate short read alignment with Burrows-Wheeler transform. *Bioinformatics* **25**, 1754-1760 (2009).

24. McKenna, A. *et al*. The Genome Analysis Toolkit: A MapReduce framework for analyzing next-generation DNA sequencing data. *Genome Research* **20**, 1297-1303 (2010).

25. Fan G, Chan J, Ma K, Yang B, Zhang H, Yang X, et al. Supporting data for "Chromosome-level reference genome of the Siamese fighting fish Betta splendens, a model species for the study of aggression" GigaScience Database 2018. http://dx.doi.org/10.5524/100433.

Figure1

Click here to access/download;Figure;Fig.1.pdf

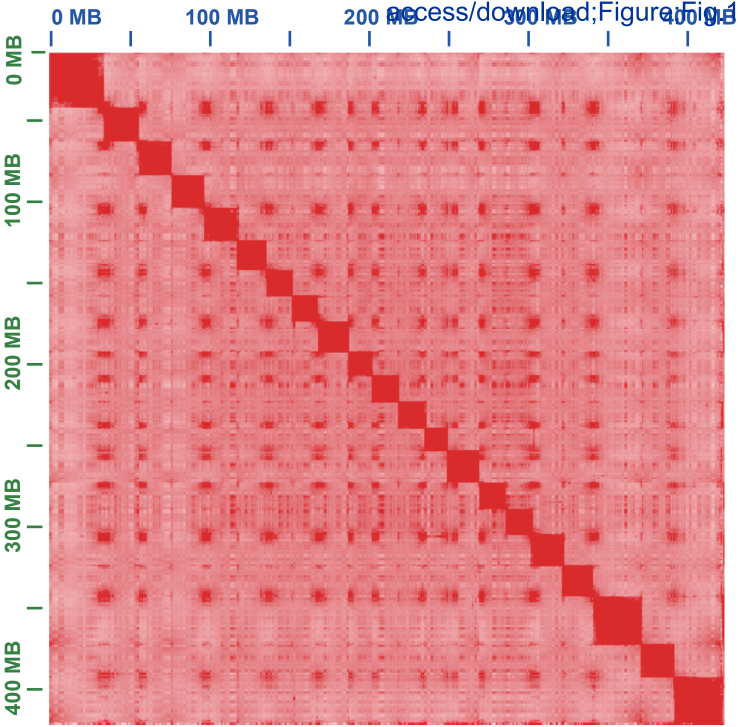

Figure2

Click here to access/download;Figure;Fig.2.png

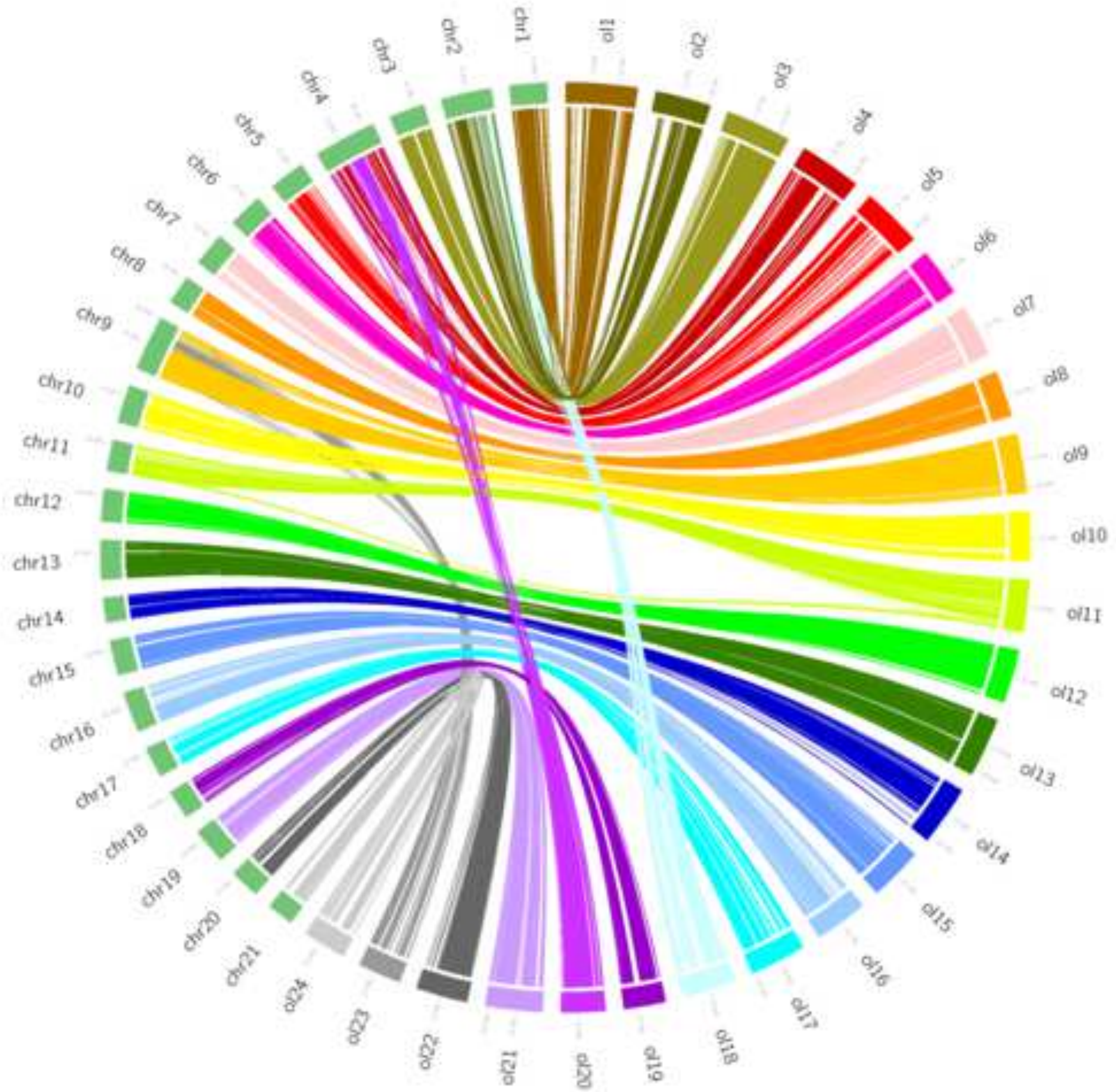

Figure3

Click here to access/download;

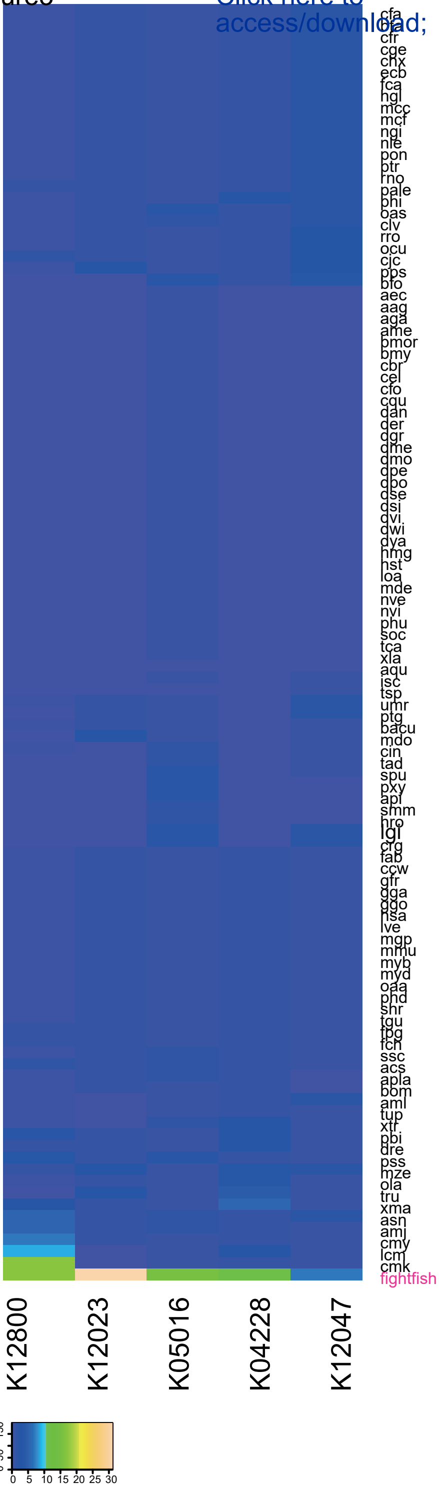

Figure4

Click here to access/download;Figure;Fig.4.pdf

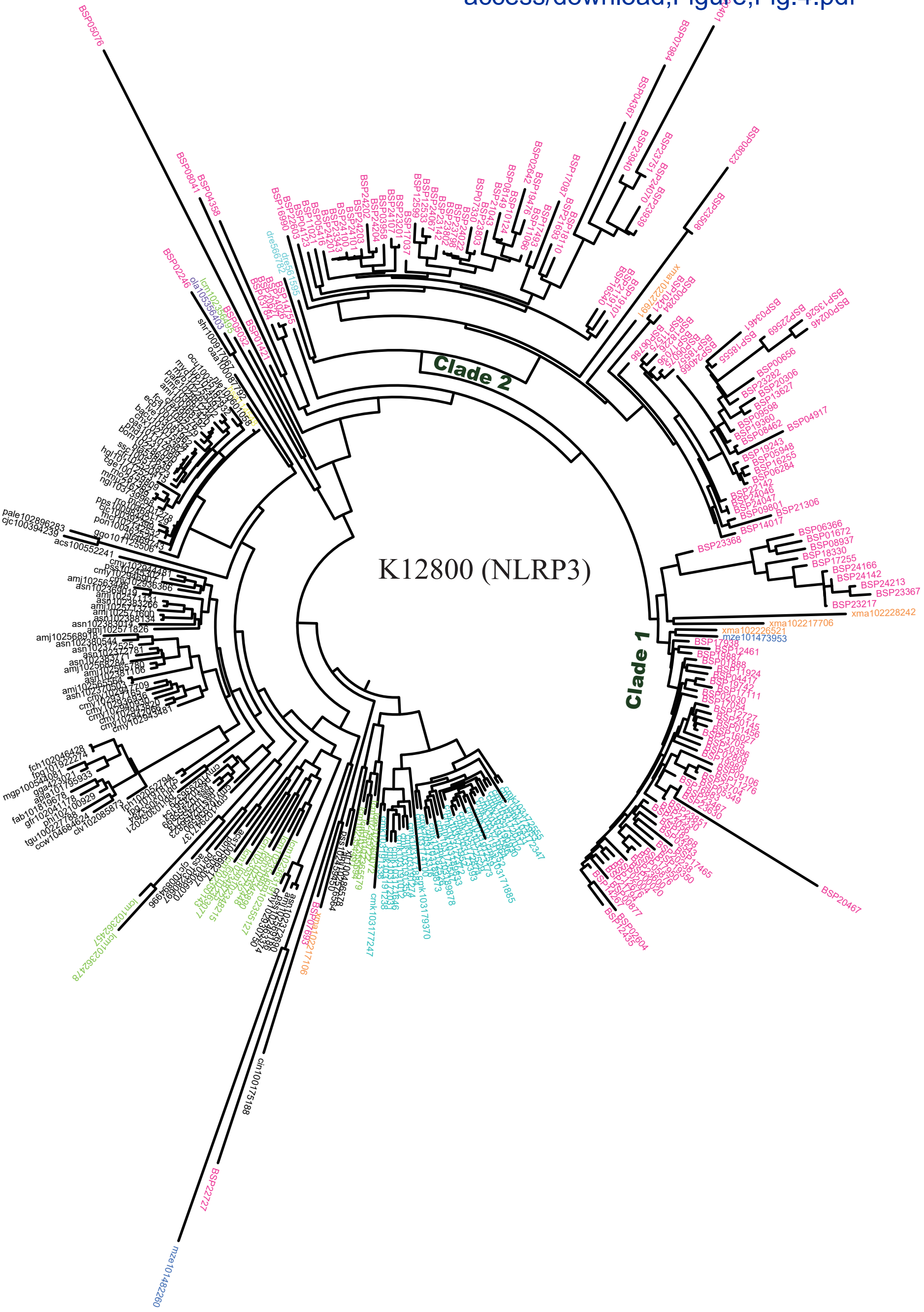

Click here to access/download
Supplementary Material
Supplementary material_revised.docx
