# Supplementary material for: Chromosome-level reference genome of the Siamese fighting fish Betta splendens, a model species for the study of aggression
Source: Gigascience. 2018 Jul 11;7(11):giy087. doi: 10.1093/gigascience/giy087 (PMC6251983; doi:10.1093/gigascience/giy087)
Supplement: Supplemental Tables and Figures [file giy087_supplemental_tables_and_figures.docx]

## Supplementary figures and tables

# Supplementary figures


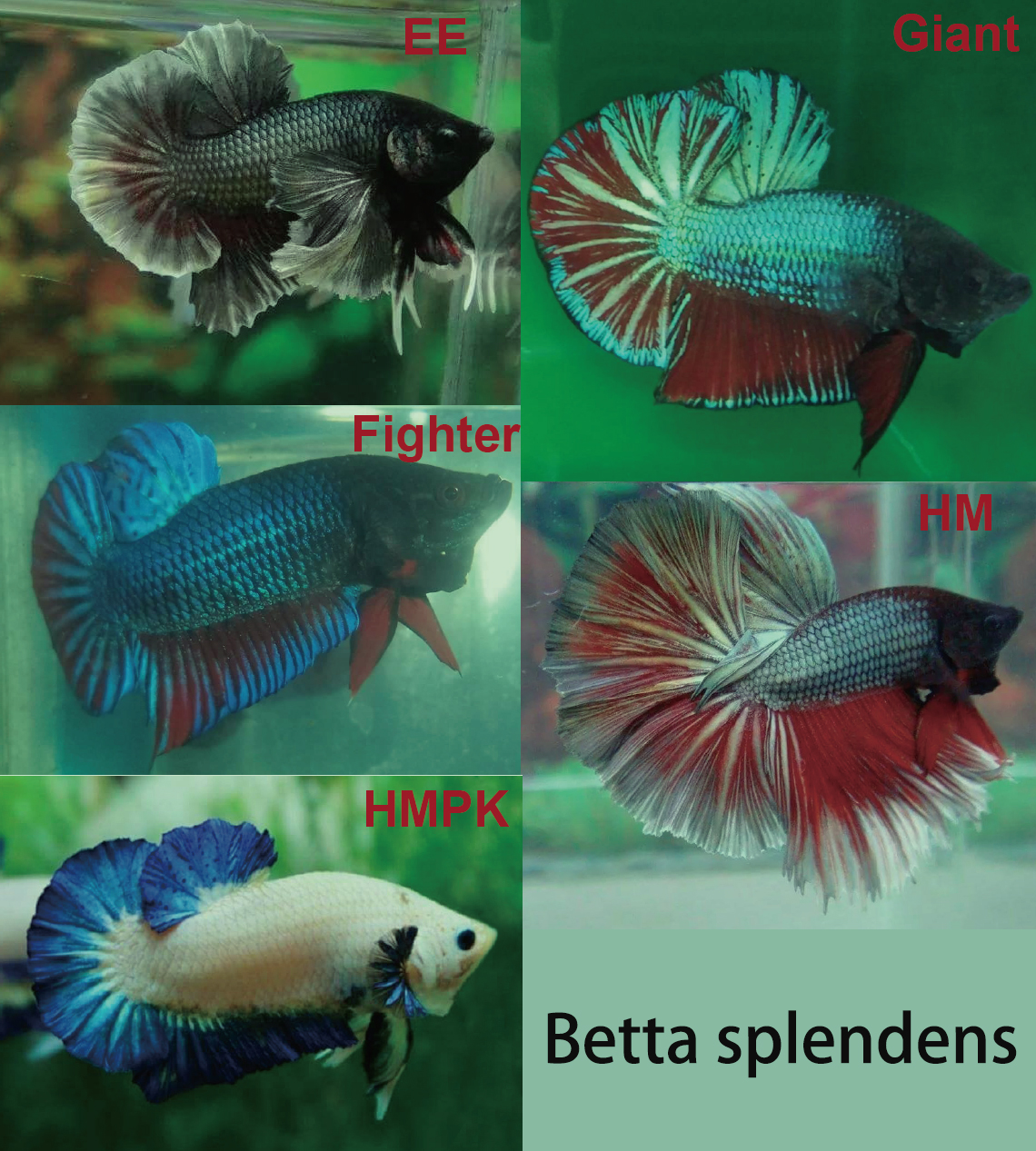


**Supplementary Fig. 1**. Five different varieties of adult male Siamese fighting fish.


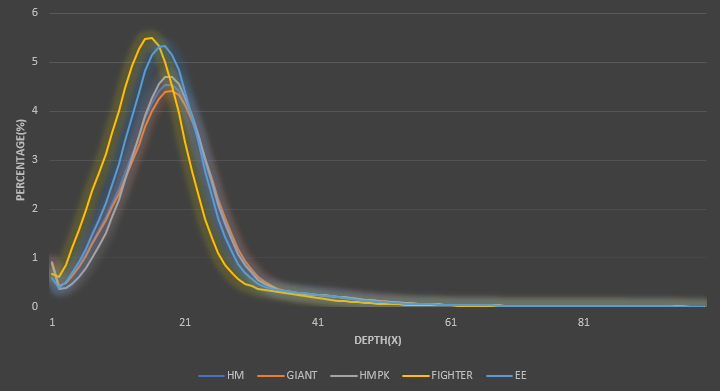


**Supplementary Fig. 2**. Distribution of the 17-mer analysis for the five Siamese fighting fish varieties.


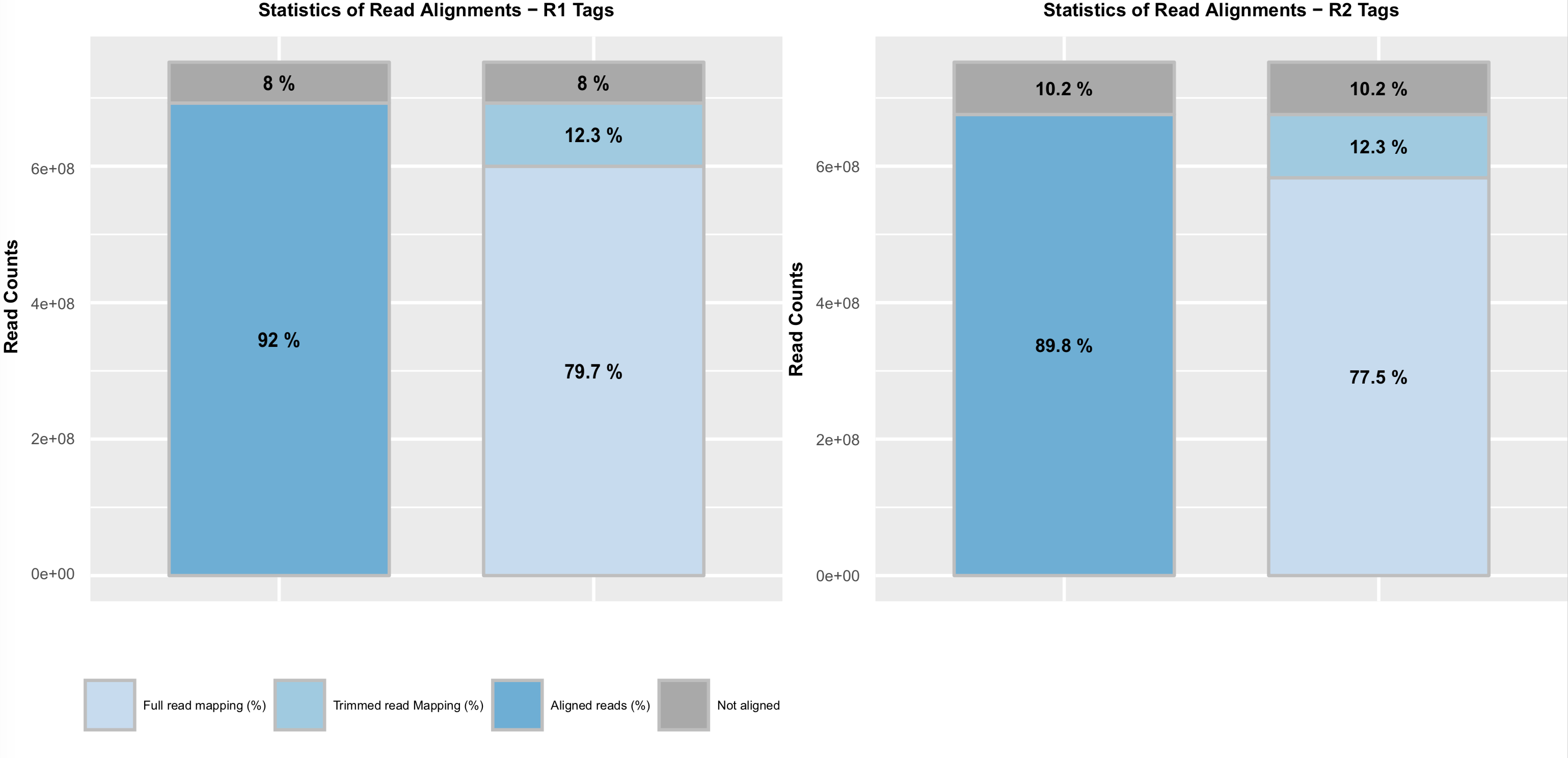


**Supplementary Fig. 3**. Quality control of Hi-C read alignment against genome sequence. Statistics for the type of separated pair-end read alignment. The aligned read ratio shown in the left bar including full read and trimmed read mapping.

**Supplementary Fig. 4**. Quality control of read pairing. Considering the alignment type and read pairing, all paired reads include uniquely aligned pairs (Reported pairs), unmapped pairs (Unmapped pairs) and others (Not Reported pairs). The right bar shows the “Not Reported pairs”, which including low quality alignment, singleton and multiple hits.


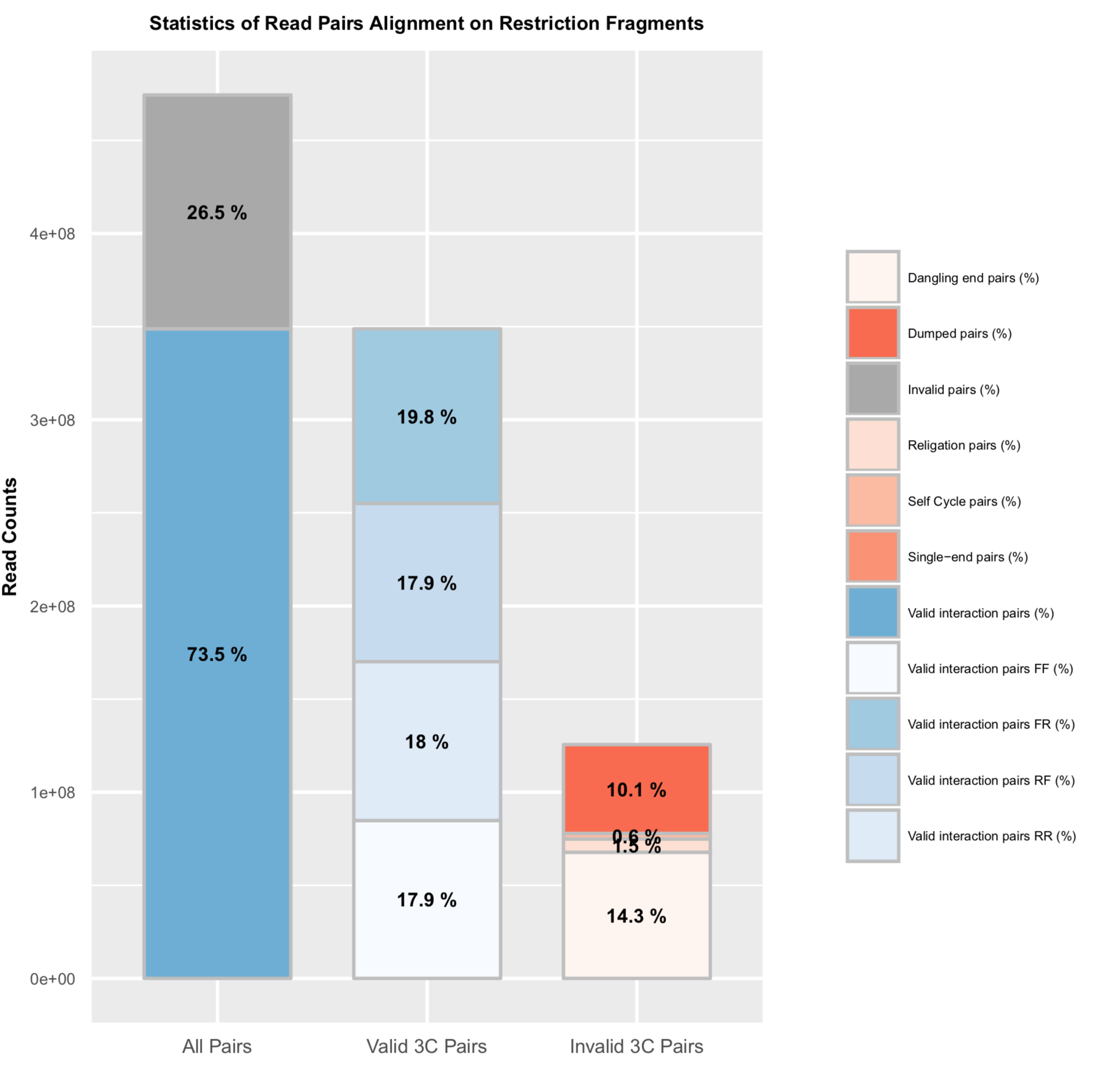


**Supplementary Fig. 5**. Statistic of read pair filtering. When assigned to restriction fragments, the aligned pairing reads can be divided to valid and invalid pairs. A valid paired-read involves two different restriction fragments and can be divided into four types according the direction of reads (the middle bar). F means Forward and R means Reverse. Invalid pairs content is shown in the right bar.


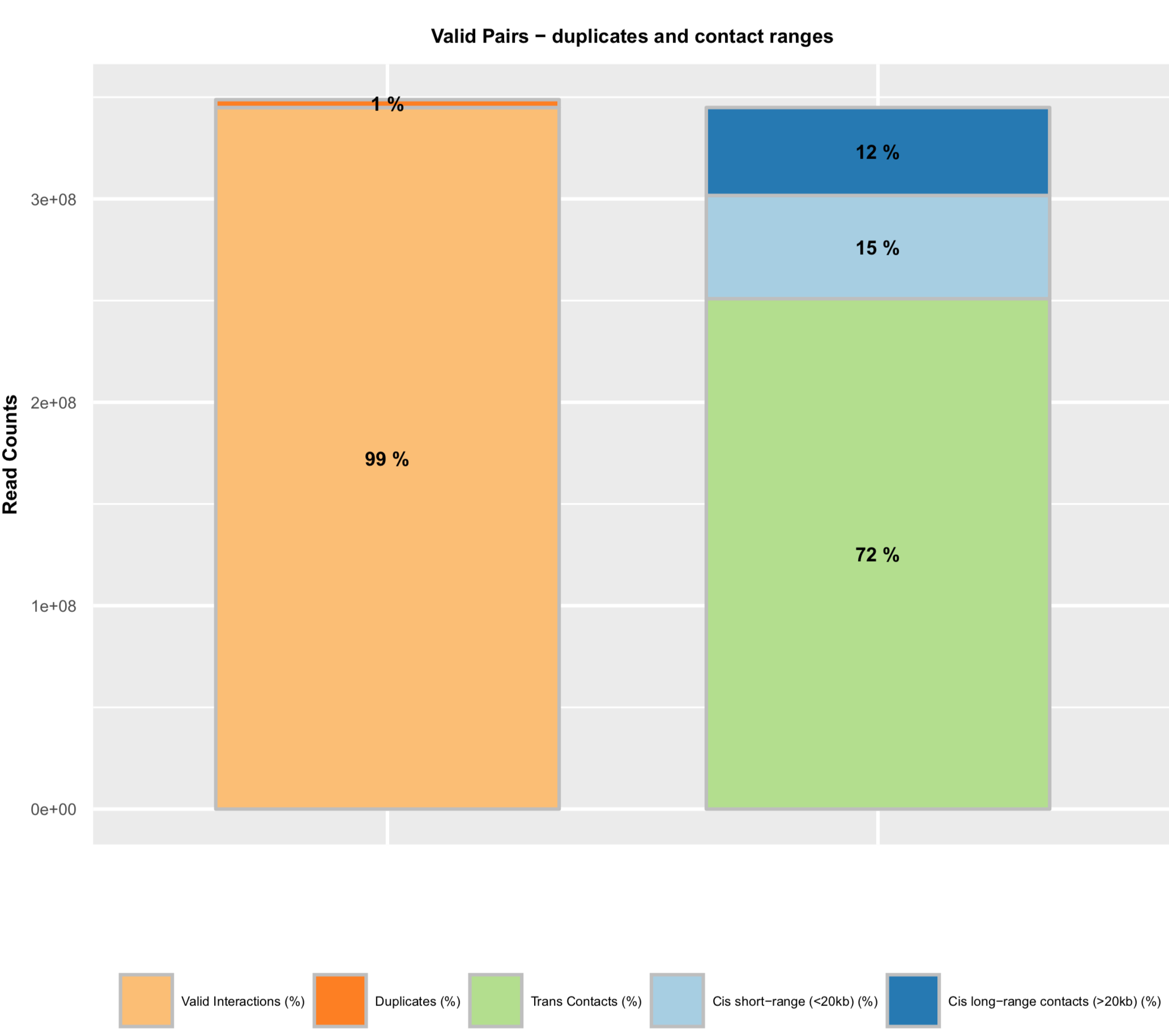


**Supplementary Fig. 6**. Fraction of duplicated reads. The left bar shows the ratio of duplication for the valid read pairs. For all the non-duplicated reads, the percentage of cis and trans contacts are shown (right bar).


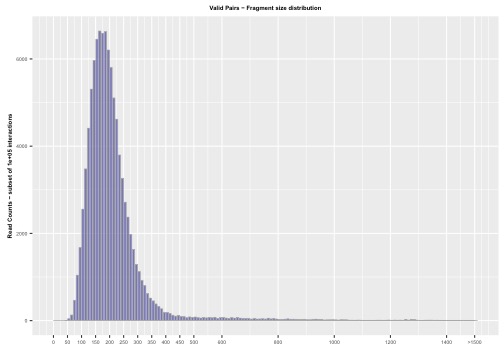


**Supplementary Fig. 7.** Distribution of the fragment size. According to the distance between alignment site and the end of restriction fragment, a fragment size can be calculated.


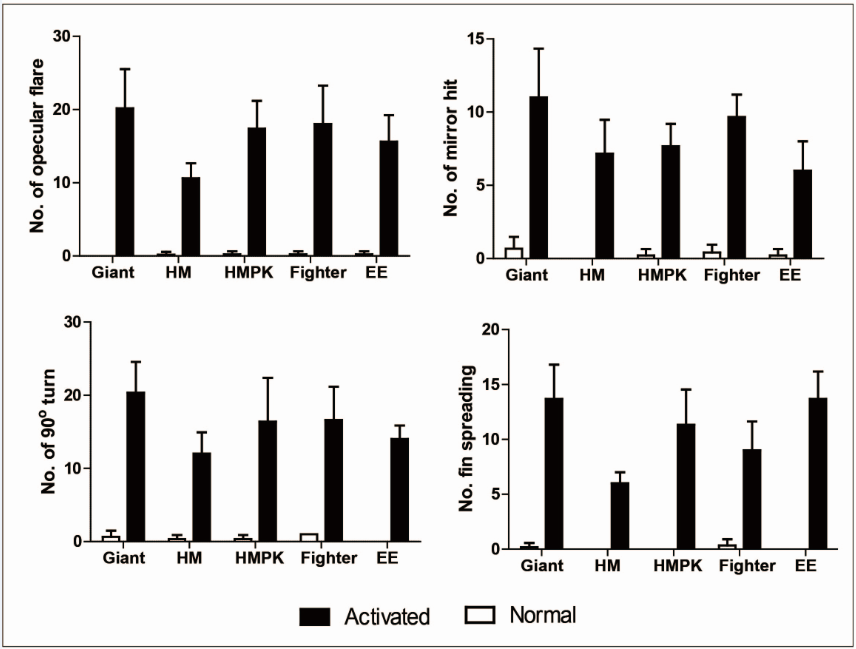


**Supplementary Fig. 8.** Evaluation of aggressive behavior in different varieties of *Betta splendens*. Number of opecular flare, mirror hit, 90º turning and fin spreading were counted manually from the 5- min video recording the swimming of normal and activated fish of different varieties. Data was expressed as mean ± S.D. of 6 replicates (n = 6).

# Supplementary tables

**Supplementary Table 1**. Summary of sequencing data generated in this study.

| Library | Reads Length | Raw Data(Gb) | Clean Data(Gb) | Raw data  Depth (X) | Clean data  Depth (X) |
| --- | --- | --- | --- | --- | --- |
| 170 | 100 | 23.00 | 21.00 | 50.00 | 45.65 |
| 500 | 100 | 19.90 | 17.50 | 43.26 | 38.04 |
| 800 | 100 | 5.60 | 4.50 | 12.17 | 9.78 |
| 2000 | 49 | 6.41 | 4.88 | 13.93 | 10.61 |
| 10000 | 49 | 11.20 | 1.90 | 24.35 | 4.13 |
| 20000 | 49 | 6.39 | 2.56 | 13.89 | 5.57 |

Note: the sequencing depth is calculated based on genome size = 0.46 Gb.

**Supplementary Table 2**. Summary of the RNA-Seq data generated using the HiSeq 2000 sequencing platform.

| Tissue | Library | Reads Length | Raw Data(Gb) | Clean Data(Gb) |
| --- | --- | --- | --- | --- |
| Brain | 150 | 90 | 7.69 | 6.22 |
| Brain-repeat | 150 | 90 | 7.34 | 5.95 |
| Liver | 150 | 90 | 7.27 | 4.83 |
| Heart | 150 | 90 | 7.74 | 5.35 |
| Muscle | 150 | 90 | 7.76 | 5.80 |

**Supplementary Table 3**. 17-mer analysis information for the five Siamese fighting fish varieties.

| **Sample** | **K-mer NO.** | **Peak**  **Depth** | **Genome Size** | **Used Bases** | **Used Reads** | **X** |
| --- | --- | --- | --- | --- | --- | --- |
| HM | 9,019,761,156 | 19 | 474,724,271 | 10,737,810,900 | 107,378,109 | 22.6 |
| GIANT | 9,133,498,332 | 20 | 456,674,916 | 10,873,212,300 | 108,732,123 | 23.8 |
| HMPK | 8,961,797,208 | 19 | 471,673,537 | 10,668,806,200 | 106,688,062 | 22.6 |
| HMPK-  FANCY | 7,758,261,840 | 17 | 456,368,343 | 9,236,026,000 | 92,360,260 | 20.2 |
| HMPK-  EE | 8,515,911,096 | 19 | 448,205,847 | 10,137,989,400 | 101,379,894 | 22.6 |

**Supplementary Table 4**. Length of the 21 chromosomes constructed for the Siamese fighting fish Giant variety, given in descending order.

| Chromosome ID | Length (bp) |
| --- | --- |
| chromosome 4 | 35,205,731 |
| chromosome 9 | 30,905,988 |
| chromosome 2 | 28,197,173 |
| chromosome 16 | 22,798,923 |
| chromosome 13 | 22,007,389 |
| chromosome 10 | 21,353,953 |
| chromosome 1 | 21,129,620 |
| chromosome 5 | 20,193,767 |
| chromosome 19 | 19,944,564 |
| chromosome 15 | 19,754,490 |
| chromosome 3 | 19,717,679 |
| chromosome 6 | 19,498,086 |
| chromosome 7 | 18,924,342 |
| chromosome 12 | 18,150,652 |
| chromosome 11 | 17,412,616 |
| chromosome 17 | 17,371,680 |
| chromosome 20 | 16,950,841 |
| chromosome 18 | 16,654,716 |
| chromosome 8 | 16,406,830 |
| chromosome 21 | 14,840,089 |
| chromosome 14 | 13,781,534 |

**Supplementary Table 5**. Assessment of the gene region coverage of assembly using RNA-seq data.

| Dataset | Number | Total  Length (bp) | Covered by Assembly (%) | With >90% Sequence in one Scaffold | | With >50% Sequence in one Scaffold | | |
| --- | --- | --- | --- | --- | --- | --- | --- | --- |
|  |  |  |  | Number | Percentage (%) | Number | | Percentage (%) |
| All | 324,627 | 341,239,189 | 99.72 | 320,091 | 98.60 | 323,814 | 99.75 | |
| >200bp | 178,893 | 321,036,273 | 99.73 | 177,429 | 99.18 | 178,627 | 99.85 | |
| >500bp | 109,428 | 300,303,774 | 99.75 | 108,852 | 99.47 | 109,310 | 99.89 | |
| >1kb | 84,138 | 282,287,383 | 99.77 | 83,796 | 99.59 | 84,075 | 99.93 | |

**Supplementary Table 6.** Statistics for the transposable element (TE) sequences present in the Siamese fighting fish genome.

|  | **RepBase TEs** | | **TE Proteins** | | ***De novo*** | | **Combined TEs** | |
| --- | --- | --- | --- | --- | --- | --- | --- | --- |
|  | **Length**  **(bp)** | **%** | **Length** | **%** | **Length**  **(bp)** | **%** | **Length**  **(bp)** | **%** |
| **DNA** | 11,367,572 | 2.69 | 1,392,071 | 0.33 | 18,482,121 | 4.38 | 23,305,568 | 5.52 |
| **LINE** | 10,419,039 | 2.47 | 8,628,640 | 2.04 | 20,479,167 | 4.85 | 24,170,420 | 5.73 |
| **LTR** | 1,031,423 | 0.24 | 0 | 0 | 2,475,607 | 0.58 | 2,898,104 | 0.68 |
| **SINE** | 7,246,291 | 1.71 | 3,835,115 | 0.90 | 16,131,592 | 3.82 | 21,145,532 | 5.01 |
| **Other** | 8,171 | 0 | 0 | 0 | 0 | 0 | 8,171 | 0.0019 |
| **Unknown** | 0 | 0 | 0 | 0 | 9,471,081 | 2.24 | 9,471,081 | 2.24 |
| **Total** | 26,372,291 | 6.25 | 13,831,796 | 3.28 | 58,498,510 | 13.87 | 63,765,075 | 15.12 |

**Supplementary Table 7.** Statistics of predicted gene models.

| **Gene set** | | **Number** | | **Average transcript length (bp)** | | **Average CDS length (bp)** | | **Average exon per gene** | | **Average exon length (bp)** | | **Average intron length (bp)** | | |  |
| --- | --- | --- | --- | --- | --- | --- | --- | --- | --- | --- | --- | --- | --- | --- | --- |
| ***De novo*** | AUGUSTUS | | 42,018 | | 5,261 | | 1,112 | | 5.81 | | 191 | | | 861 | |
|  | GENSCAN | | 31,764 | | 9,652 | | 1,696 | | 8.88 | | 190 | | | 1,008 | |
| **GLEAN Final set** | | 23,981 | | 6,421.71 | | 1,626.98 | | 8.9 | | 182.65 | | | 606.37 | |  |

**Supplementary Table 8.** Summary of the alignment between *Betta splendens* and *Oryzias latipes*

| *Betta splendens* chromosome ID | Best *Oryzias latipes* chromosome | Best coverage | Second *Oryzias latipes* chromosome | Second coverage |
| --- | --- | --- | --- | --- |
| chromosome 4 | 20 | 43.94% | 4 | 37.84% |
| chromosome 3 | 3 | 85.19% | ultracontig49 | 1.31% |
| chromosome 8 | 8 | 83.10% | 1 | 1.28% |
| chromosome 14 | 14 | 79.87% | scaffold223 | 2.64% |
| chromosome 13 | 13 | 78.25% | 4 | 1.06% |
| chromosome 20 | 22 | 77.92% | 24 | 1.95% |
| chromosome 7 | 7 | 76.49% | 5 | 2.33% |
| chromosome 6 | 6 | 75.99% | ultracontig72 | 1.19% |
| chromosome 15 | 15 | 74.06% | 1 | 1.13% |
| chromosome 17 | 17 | 73.38% | 4 | 1.97% |
| chromosome 19 | 21 | 72.60% | ultracontig257 | 3.67% |
| chromosome 10 | 10 | 70.10% | ultracontig115 | 6.50% |
| chromosome 1 | 1 | 69.71% | scaffold344 | 1.71% |
| chromosome 12 | 12 | 68.53% | 14 | 3.46% |
| chromosome 21 | 24 | 65.94% | 16 | 1.36% |
| chromosome 16 | 16 | 63.61% | ultracontig182 | 8.65% |
| chromosome 18 | 19 | 63.52% | 9 | 4.93% |
| chromosome 5 | 5 | 63.27% | ultracontig62 | 6.42% |
| chromosome 9 | 9 | 62.41% | 23 | 19.36% |
| chromosome 11 | 11 | 59.31% | 17 | 1.87% |
| chromosome 2 | 18 | 35.08% | 2 | 28.96% |

**Supplementary Table 9**. Summary of the resequencing data generated in this study.

| variations | Library | Reads  Length | Raw  Data(Gb) | Clean  Data(Gb) | Raw Data  Depth(X) | Clean Data  Depth(X) |
| --- | --- | --- | --- | --- | --- | --- |
| HM | 500 | 100 | 20.97 | 18.53 | 45.58 | 40.28 |
| HMPK | 500 | 100 | 20.93 | 18.36 | 45.5 | 39.91 |
| HMPK-FANCY | 500 | 100 | 17.87 | 15.93 | 38.84 | 34.63 |
| HMPK-EE | 500 | 100 | 19.41 | 17.43 | 42.19 | 37.89 |
|  | Total | -- | 79.18 | 70.25 | 172.11 | 152.71 |

Note: the sequencing depth is calculated based on genome size = 0.46 Gb.
