# Supplementary material for: Chromosome-level reference genome of the Siamese fighting fish Betta splendens, a model species for the study of aggression
Source: Gigascience. 2018 Jul 11;7(11):giy087. doi: 10.1093/gigascience/giy087 (PMC6251983; doi:10.1093/gigascience/giy087)
Supplement: Response_to_Reviewer_Comments_Original_Submission.pdf [file giy087_response_to_reviewer_comments_original_submission.pdf]

Author's Response To Reviewer Comments

Close

We are very much thankful to you handling this MS as well as the acceptance of our MS for publication in GigaScience. We are also thankful to you and two reviewers for appreciation of our work. We addressed all the comments of two reviewers, as a result, we have updated the gene set,several main text and supplementary files. As suggested, we have prepared point-by-point response to each concern/comment. We have uploaded the revised version of the MS, the response to the comments, as well as the process results files into GigaDB.We uploaded a protocol file and a One-to-one-response-letter.

Close

.
