# Supplementary material for: Chromosome-level reference genome of the Siamese fighting fish Betta splendens, a model species for the study of aggression
Source: Gigascience. 2018 Jul 11;7(11):giy087. doi: 10.1093/gigascience/giy087 (PMC6251983; doi:10.1093/gigascience/giy087)
Supplement: Response_to_Reviewer_Comments_Revision_1.pdf [file giy087_response_to_reviewer_comments_revision_1.pdf]

Author's Response To Reviewer Comments

Close

Dear edit,

We are very grateful to you and the reviewers for re-review our submission.We are honored that our manuscript can be accepted for publication in GigaScience.We made changes based on your suggestions and responded point-by-point.

1. Please include the version numbers for all software tools you used.
Answer: We have added the version number of the software tool.

2. Thank you for using protocols.io, to share protocols, that's great. Please include the protocols in your bibliography and cite them in your text where appropriate. For example, when referring to the DNA extraction method in the manuscript, you should cite your respective protocol: [xx] Ma K. DNA extraction for the Betta splendens genome. Protocols.io. 2018. http://dx.doi.org/10.17504/protocols.io.qvedw3e ... and the same way for your other protocols (please note that each protocol has an individual doi number).
Answer: We have added the protocol to the bibliography and cite them in my text where appropriate.

3. Please mention the fish base ID for the species in the manuscript
Answer: We added the NCBI Taxonomy ID to identify this species.

4. I included a citation to your GigaDB dataset in the data availability section, please double check.
Answer: Thank you for your help in this section. We have updated a citation to my GigaDb dataset at the bottom of the text.

5. I resolved some "tracked changes" in the attached version of the manuscript, please double check this is now the correct version.
Answer: Thank you for helping us solve the "tracked changes". We have checked it again.

6.Please check your supplementary material - I think there are still some "tracked changes" that need to be resolved.
Answer: We checked and solved the "tracked changes" in the supplementary material

7. I included some Research Resource Identifiers (RRIDs) for software tools you used - please double check.
Answer: Thank you for your help in this section.I checked the Research Resource Identifiers you added and added the RRID of the other software tools.

Close
