# Supplementary material for: Chromosome-level reference genome of the Siamese fighting fish Betta splendens, a model species for the study of aggression
Source: Gigascience. 2018 Jul 11;7(11):giy087. doi: 10.1093/gigascience/giy087 (PMC6251983; doi:10.1093/gigascience/giy087)
Supplement: Reviewer_1_Report_(Original_Submission) -- Ole K Tørresen — 3/13/2018 Reviewed [file giy087_reviewer_1_report_(original_submission).pdf]

**Reviewer Report**

**Title: Chromosome-level reference genome of the Siamese fighting fish Betta splendens, a model species for the study of aggression**

**Version: Original Submission** **Date:** 3/13/2018

**Reviewer name: Ole K Tørresen**

**Reviewer Comments to Author:**

Title: High-quality reference genome of the Siamese fighting fish Betta splendens, a model species for the study of aggression ## General comments ##The authors have produced a genome assembly for the Siamese fighting fish, the Giant variety. This fish is known for it's aggressive behavior and is a model species for investigating this trait. The authors do not state whether or not the different varieties differ in aggressiveness, but if they do, that would be an obvious angle for investigating this trait. Quality is always difficult to measure, but one way is to find something all agree is of good quality and compare against that. In this day of long-read sequencing based genome assemblies, I find it problematic to state that something is of high quality when it is more fragmented than other genome assemblies of fish. For instance, the genome assembly of the orange clownfish was recently reported at bioRxiv (https://www.biorxiv.org/content/early/2018/03/07/278267), and it contain both longer contigs and scaffolds, and more genes found with BUSCO (but the actinopterygii gene set and not the vertebrate). They state that their genome assembly is of high quality, and I concur with their evaluation of their assembly. The fighting fish genome assembly is of lesser quality than the orange clownfish. However, it is worthy of publication, but maybe with a bit more humble language.There is a lack of commands and settings used for the different programs in this manuscript. Optimally, a manuscript should have enough detail so the analysis can be reproduced. This is often difficult, but then there should be more details so it is possible to better understand what has been done. At a minimum, and you should always strive to do better than a minimum, you should provide the versions of the different programs used. ## Specific comments ##Lines 28, 29, 79 and 151: Shouldn't kilobases be abbreviated to kb, not Kb? It should, at least according to https://en.wikipedia.org/wiki/Metric_prefix. Line 37: As far as I can understand, the "15% repetitive sequences" referred to here is just transposable elements and not simple/tandem repeats. Either include the simple repeats content also or state that it is only transposable elements.Line 63: What does 'HK supplier' means in this context? Hong Kong?Line 73: Please specify these settings for SOAPnuke.Line 75: Please specify the settings for SOAPdenovo if they differ from default settings.Line 78: Also specify settings for GapCloser (you have it misspelled with a small case c) if they differ from defaults.Line 78: It is more accurate to state that you obtained a 'genome assembly', not a genome.Line 80: Would you expect this result, that the assembly is almost exactly the size of the estimation? I would guess that the assembly lacks the telomeres and centromeres and therefore are smaller than the true size of the genome. It seems that the estimation then would not take the sequences found in the telomeres and centromeres into account, and it is therefore an underestimation of the true genome size.Lines 86 and 87: Really interesting to see that the Hi-C method works so well in reconstructing the chromosomes.Line 89: What are the BUSCO scores of the original assembly? It would be interesting to see the improvement from placing the scaffolds into a chromosomal context. Also, why do you use the vertebrate gene set and not the actinopterygii specific set?Line 102: Shouldn't Augustus be with capital letters?Line 104: Why didn't you use all five RNA-seq libraries?Lines 108 and Table 2: Why was the vertebrate gene set used and not the Actinopterygii? The Actinopterygii gene set is specific to the ray-finned fishes, and would be more suitable and better for this fish.Lines 114-115: Which program and settings were used to do this comparison?Table 1: There is a big difference between the N50 contig lengths of the original and the Hi-C assembly, actually an order of magnitude. Why this big difference? Could you comment on it in the manuscript? Did you run GapCloser on the Hi-C assembly? Please state it if you did. Also, the amount of bases in contigs went substantially down (about 12 Mbp). Do you have any idea why? The bases scaffolds also went substantially down. Did you throw out short contigs/scaffolds?Table 2: As mentioned above: Why did you use the vertebrate gene set

and not the actinopterygii? You should also mention that you used the vertebrate gene set explicitly.Figure 3: Figure text should be self-explanatory. Please expand this and for instance state which gene families these are. I know you have in the main text, but it is good to repeat it here or at least refer to the main text.Supplementary Table 2: Here you have only four tissues, but you state in line 66 that you have five RNA-seq libraries. However, I guess that five is wrong, because you state four in the abstract.Supplementary Table 4: What is the ID of the chromosome based on here? Often the largest chromosome is called 'chromosome 1'. This is not the case here, so I presume that you base the IDs on something else. Please state what that is.

**Level of Interest**

Please indicate how interesting you found the manuscript: An article whose findings are important to those with closely related research interests

**Quality of Written English**

Please indicate the quality of language in the manuscript: Acceptable

**Declaration of Competing Interests**

Please complete a declaration of competing interests, considering the following questions:

- Have you in the past five years received reimbursements, fees, funding, or salary from an organisation that may in any way gain or lose financially from the publication of this manuscript, either now or in the future?
- Do you hold any stocks or shares in an organisation that may in any way gain or lose financially from the publication of this manuscript, either now or in the future?
- Do you hold or are you currently applying for any patents relating to the content of the manuscript?
- Have you received reimbursements, fees, funding, or salary from an organization that holds or has applied for patents relating to the content of the manuscript?
- Do you have any other financial competing interests?
- Do you have any non-financial competing interests in relation to this paper?

If you can answer no to all of the above, write 'I declare that I have no competing interests' below. If your reply is yes to any, please give details below.

I declare that I have no competing interests

I agree to the open peer review policy of the journal. I understand that my name will be included on my report to the authors and, if the manuscript is accepted for publication, my named report including any attachments I upload will be posted on the website along with the authors' responses. I agree for my report to be made available under an Open Access Creative Commons CC-BY license (http://creativecommons.org/licenses/by/4.0/). I understand that any comments which I do not wish to

be included in my named report can be included as confidential comments to the editors, which will not be published.

I agree to the open peer review policy of the journal

To further support our reviewers, we have joined with Publons, where you can gain additional credit to further highlight your hard work (see: https://publons.com/journal/530/gigascience). On publication of this paper, your review will be automatically added to Publons, you can then choose whether or not to claim your Publons credit. I understand this statement. Yes
